# *Pantoea* Bacteriophage vB_PagS_MED16—A Siphovirus Containing a 2′-Deoxy-7-amido-7-deazaguanosine-Modified DNA

**DOI:** 10.3390/ijms22147333

**Published:** 2021-07-08

**Authors:** Monika Šimoliūnienė, Emilija Žukauskienė, Lidija Truncaitė, Liang Cui, Geoffrey Hutinet, Darius Kazlauskas, Algirdas Kaupinis, Martynas Skapas, Valérie de Crécy-Lagard, Peter C. Dedon, Mindaugas Valius, Rolandas Meškys, Eugenijus Šimoliūnas

**Affiliations:** 1 Department of Molecular Microbiology and Biotechnology, Institute of Biochemistry, Life Sciences Centre, Vilnius University, Saulėtekio av. 7, LT-10257 Vilnius, Lithuania; monika.simoliuniene@gmc.vu.lt (M.Š.); emilija.zukauskiene@bchi.stud.vu.lt (E.Ž.); lidija.truncaite@bchi.vu.lt (L.T.); rolandas.meskys@bchi.vu.lt (R.M.); 2 Singapore-MIT Alliance for Research and Technology, Antimicrobial Resistance Interdisciplinary Research Group, Campus for Research Excellence and Technological Enterprise, Singapore 138602, Singapore; liangcui@smart.mit.edu (L.C.); pcdedon@mit.edu (P.C.D.); 3 Department of Microbiology and Cell Science, University of Florida, Gainesville, FL 32611, USA; ghutinet@ufl.edu (G.H.); vcrecy@ufl.edu (V.d.C.-L.); 4 Department of Bioinformatics, Institute of Biotechnology, Life Sciences Centre, Vilnius University, Saulėtekio av. 7, LT-10257 Vilnius, Lithuania; darius.kazlauskas@bti.vu.lt; 5 Proteomics Centre, Institute of Biochemistry, Life Sciences Centre, Vilnius University, Saulėtekio av. 7, LT-10257 Vilnius, Lithuania; algirdas.kaupinis@gf.vu.lt (A.K.); mindaugas.valius@bchi.vu.lt (M.V.); 6 Department of Characterisation of Materials Structure, Center for Physical Sciences and Technology, Saulėtekio av. 3, LT-10257 Vilnius, Lithuania; martynas.skapas@ftmc.lt; 7 Genetics Institute, University of Florida, Gainesville, FL 32610, USA; 8 Department of Biological Engineering and Center for Environmental Health Sciences, Massachusetts Institute of Technology, Cambridge, MA 02139, USA

**Keywords:** *Pantoea* *agglomerans*, bacteriophage, vB_PagS_MED16, siphovirus, DNA modifications, DpdA, 2′-deoxy-7-amido-7-deazaguanosine (dADG)

## Abstract

A novel siphovirus, vB_PagS_MED16 (MED16) was isolated in Lithuania using *Pantoea agglomerans* strain BSL for the phage propagation. The double-stranded DNA genome of MED16 (46,103 bp) contains 73 predicted open reading frames (ORFs) encoding proteins, but no tRNA. Our comparative sequence analysis revealed that 26 of these ORFs code for unique proteins that have no reliable identity when compared to database entries. Based on phylogenetic analysis, MED16 represents a new genus with siphovirus morphology. In total, 35 MED16 ORFs were given a putative functional annotation, including those coding for the proteins responsible for virion morphogenesis, phage–host interactions, and DNA metabolism. In addition, a gene encoding a preQ_0_ DNA deoxyribosyltransferase (DpdA) is present in the genome of MED16 and the LC–MS/MS analysis indicates 2′-deoxy-7-amido-7-deazaguanosine (dADG)-modified phage DNA, which, to our knowledge, has never been experimentally validated in genomes of *Pantoea* phages. Thus, the data presented in this study provide new information on *Pantoea*-infecting viruses and offer novel insights into the diversity of DNA modifications in bacteriophages.

## 1. Introduction

Bacteriophages, the viruses that infect bacteria, are the most abundant biological entities in the biosphere [1]. Phages are widespread in a variety of environments and play a major role in modulating the abundance of bacterial populations. Over years of evolution, bacteria have developed mechanisms of defending themselves from phages, such as the use of CRISPR-Cas and restriction-modification systems [2,3]. On the other hand, phages have evolved counter measures to escape these defenses, and one of the most widespread strategies is to modify their DNA [4,5,6].

Collectively, bacteriophages contain the greatest diversity of known naturally occurring modified DNA bases compared with any group of cellular organisms [7,8,9,10]. Non-canonical deoxyribonucleosides have been reported for all four bases of phage genomic DNAs, for example, 5-methyl-2′-deoxycytidine (m5dC) in *Xanthomonas* phage XP12 [11,12], 2′-deoxy-7-formamidimide-7-deazaguanosine (dG^+^) in Enterobacteria phage 9g [13,14], *N*^6^-methyl-2′-deoxyadenosine (m6dA) in *Escherichia* phage T2 [15], and 5-putrescinylthymidine (α-putT) in *Pseudomonas* phage phi W-14 [16,17]. Moreover, although several new base modifications were recently discovered in the genomes of tailed phages [9,13,14,18,19,20,21], it seems that the full extent of possible DNA modifications of these viruses is still underexplored.

Five genomic guanosine modifications have been recently identified in phage DNA by combining in silico data mining and experimental validation [13,14,18,19]. It has been demonstrated that 2′-deoxyguanosine (dG) residues are replaced by: 2′-deoxy-7-formamidimide-7-deazaguanosine (dG^+^) in Enterobacteria phage 9g [13,14], Halovirus HVTV-1 and *Vibrio* phage nt-1 [18]; 2′-deoxy-7-aminomethyl-7-deazaguanosine (dPreQ_1_) in Halovirus HVTV-1 and *Streptococcus* phage Dp-1 [18]; 2′-deoxy-7-cyano-7-deazaguanosine (dPreQ_0_) in *Escherichia* phage Cajan, *Mycobacterium* phage Rosebush and *Vibrio* phage nt-1 [18]; 2′-deoxy-7-amido-7-deazaguanosine (dADG) in Halovirus HVTV-1, *Salmonella* phage 7–11, *Vibrio* phage nt-1 [18], *Campylobacter* phages CP220 and CPt10 [19]; and deoxyinosine (dI) in *Campylobacter* phages NCTC 12673, NCTC 12,669 and F336 [19]. To our knowledge, none of the aforementioned guanosine hypermodifications have been experimentally validated in the genomes of *Pantoea* bacteriophages.

In this study, we report the experimental identification of hypermodified guanosine nucleotides in the DNA of two *Pantoea agglomerans*-infecting phages: vB_PagS_MED16 (referred to by its shorter name, MED16, below) and vB_PagS_Vid5 (Vid5) [22]. LC–MS/MS analysis indicates that dADG and dG+ are present in the genomes of MED16 and Vid5, respectively. Additionally, here we describe a complete genome analysis and the biological characteristics of lytic bacteriophage MED16, which shows a low-temperature plating profile and forms plaques even at 4 °C. Phylogenetic analysis indicates that phage has no close phylogenetic relationship with other bacteriophages and potentially forms a new genus within the siphoviruses. Thus, the data presented here not only provide information on morphology, physiology, and genetic diversity of *Pantoea*-infecting viruses, but also broaden our understanding about guanosine hypermodifications in bacteriophages.

## 2. Results

### 2.1. Phage Morphology, Host Range and Physiological Characteristics

Transmission electron microscopy (TEM) analysis revealed that MED16 is a siphovirus that corresponds to the B1 morphotype in Bradley’s classification [23,24]. The phage MED16 is characterized by an isometric head (61.03 ± 2.04 (n = 25)) and an apparently non-contractile flexible tail (157.63 ± 9.89 (n = 24) nm in length, and 10.44 ± 1.19 (n = 25) in width). Notably, short tail fibers attached to a distal part of the tail of MED16 were visible using TEM.

**Figure 1:**
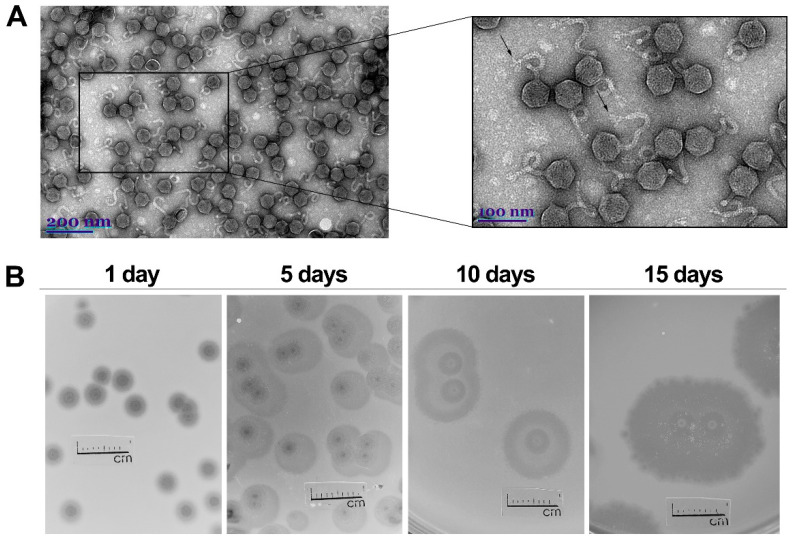
Electron micrographs of CsCl-purified MED16 particles (**A**) and morphology of plaques formed by phage MED16 on a lawn of *Pantoea agglomerans* strain BSL (**B**). (**A**) Black arrows indicate tail fibers; (**B**) plates were incubated at 22 °C, numbers above indicate days of incubation.

In total, 24 bacterial strains closely related by clades (Supplementary Table S1) were used to explore the host range of bacteriophage MED16. With the exception of *Pantoea agglomerans* strain BSL, the remaining six *Pantoea* spp. isolates, as well as all the tested strains of *Acinetobacter*, *Citrobacter*, *Erwinia*, *Escherichia*, *Klebsiella*, *Salmonella*, and *Pseudomonas* spp., were found to be not infected by MED16. To determine the optimal conditions for phage propagation, the effect of temperature on the efficiency of plating (e.o.p.) was examined in the temperature range of 3–40 °C. The test revealed that MED16 forms plaques at 4–36 °C and is at the optimum temperature for plating, about 30 °C (Supplementary Figure S1). Therefore, based on the characteristics of physiological types of bacteriophages recognized by Seeley and Primrose [25], phage MED16 belongs to the group of low-temperature phages, which are active at or below 30 °C. Moreover, the results of the (e.o.p.) experiments indicate that MED16 could be a good example of phages adapted to infect their hosts at ambient temperatures. The plaques of MED16 have a clear center surrounded by constantly growing opaque halo zones (Figure 1B), indicating the presence of phage-encoded bacterial exopolysaccharide (EPS)-degrading depolymerases, which usually act either as integral components of the virion particles or as free soluble enzymes [26]. After one day of incubation at 22 °C, the plaques formed by MED16 are up to 4.19 ± 0.37 mm in diameter, after five days, 7.21 ± 0.69 in diameter, after ten days, 12.89 ± 0.27 in diameter, and the plaques reach 18.69 ± 0.34 mm in diameter within a period of 15 days. The adsorption test showed that a high percentage (~85%) of the MED16 particles adsorb *Pantoea agglomerans* strain BSL after the first 2 min of incubation, and, after 15 min, only ~1% of the input virions remained unattached (Supplementary Figure S2). The high value of non-adsorbed phage particles at 30 min could be explained by the appearance of phage progenies. However, additional experiments indicating the latent and eclipse periods of MED16 needs to be performed to confirm this hypothesis.

### 2.2. Overview of Genome

**Figure 2:**
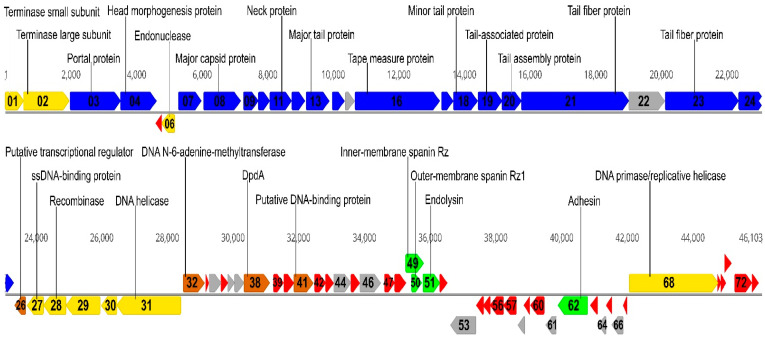
Functional genome map of bacteriophage MED16. The coding capacity of the genome is shown. Numbers indicate ORF position in genome; functions are assigned according to the characterized ORFs in NCBI database and HHpred analysis. The color code is as follows: yellow—DNA replication, recombination, repair and packaging; blue—structural proteins; brown—transcription, translation, nucleotide metabolism; green—lysis, phage–host interaction; grey—conserved hypothetical proteins; red—MED16 hypothetical proteins with no reliable identity when compared to database entries.

Phage MED16 has a linear, double-stranded DNA genome consisting of 46,103 bp with a G-C content of 55.1%, which is similar to that (52%–55%) observed for *Pantoea* spp. [27]. Like those of other dsDNA bacteriophages, the genome of MED16 is close-packed—96.4% of the genome is coding. The analysis of the genome sequence revealed that MED16 has 73 predicted open reading frames encoding for only proteins, but no tRNAs (Figure 2; Supplementary Table S2). Notably, an apparent asymmetry in the distribution of the genes on the two DNA strands of phage was observed. In total, 50 ORFs of MED16 are predicted to be transcribed from the same DNA strand, while the other 23 ORFs are found on the opposite DNA strand.

Bioinformatics analysis revealed that 26 out of 73 MED16 ORFs encode unique proteins that have no reliable identity (E-values > 0.001) when compared to NCBI nr entries. In the case of MED16 ORFs that encode proteins with matches to those in other sequenced genomes, the percentage of amino acid identity ranged from 28% to 84% and, in most cases (37 out of 47 ORFs), this was from 36% to 55% (Supplementary Table S2). Among the MED16 gene products with detectable homologues in other sequenced genomes, 46 have the best E-values to proteins from phages that infect *Cronobacter, Dickea, Edwardsiella, Enterobacter, Escherichia*, *Klebsiella*, *Pantoea, Pseudomonas*, *Salmonella*, *Xanthomonas*, and *Yersinia*. One MED16 gene product (hypothetical protein encoded by ORF61) is similar to proteins found in bacteria exclusively, and it has the best match with a hypothetical protein (ORM93120.1) from *Pantoea cypripedii* (Supplementary Table S2). Based on homology to biologically defined proteins, 35 ORFs of MED16 were given a putative functional annotation, including those coding proteins responsible for virion morphogenesis, phage–host interactions, and DNA metabolism. As was observed in other siphoviruses, the genome of MED16 appears to have a modular organization, with genes for DNA packaging, structure/morphogenesis, host lysis, replication/regulation and nucleotide metabolism clustered together (Figure 2). Notably, none of the predicted gene products show sequence homology with integration-related proteins or antibiotic resistance determinants.

### 2.3. Structural Proteins

Bioinformatics analysis of the genome sequence of bacteriophage MED16 allowed the identification of 17 structural genes, including those coding for the head (ORF03, ORF04, ORF07–ORF10), tail (ORF11–ORF14, ORF16–ORF20), and tail fiber (ORF21, ORF23) proteins (Supplementary Table S2). The major capsid protein (MCP) and major tail protein (MTP) are two of the main building components constituting the virions of siphoviruses [28]. The major capsid protein (gp08) of MED16 exhibits the highest similarity to major capsid proteins from a variety of Enterobacteria-infecting bacteriophages. The HHpred analysis revealed that MED16 gp08 best matches the structure of the major capsid protein of *Bacillus* phage SPP1 (6R3A_G; probability, 100%; E-value, 3.2 × 10^−33^). The major tail protein (gp13) of MED16 belongs to the Phage_tail_3 (cl07426) superfamily and has the best HHpred hit to the major tail protein of Enterobacteria phage lambda (2K4Q_A; probability, 99.57%; E-value, 1.1 × 10^−14^).

As mentioned above, the short tail fibers of MED16 were clearly visible by TEM (Figure 1). Accordingly, two MED16 genes coding for tail fiber proteins (gp21 and gp23) were identified by bioinformatics approaches. The tail fiber protein encoded by ORF21 contains conserved COG4733 (COG4733), DUF1983 (pfam09327) and FN3 (smart00060) domains, whereas the tail fiber protein encoded by ORF23 has a conserved pyocin_knob (cd19958) domain and Pectate_lyase_3 (pfam12708) domain, which is commonly found in pectin/pectate lyases of bacteriophages [26]. The gp24 shares the highest similarity to hypothetical proteins of *Pantoea* and *Erwinia* phages (with 41% aa sequence identity and an E-value of 1 × 10^−43^; the best BLASTp hit is a hypothetical protein (gp48) of *Pantoea* phage vB_PagM_LIET2). Although no conserved domains were detected in gp24 of MED16 and its closest homologues, based on the position in the genome (it is located just downstream the gp23), homology to publicly available proteins, and results of proteomics analysis, it is likely that gp24 of MED16 may play important role in phage–host interactions.

FASP followed by LC–MS/MS confirmed eight MED16 structural proteins identified by comparative genomics and/or HMM profile comparisons (Table S3). It was confirmed that gp03 (portal protein), gp04 (head morphogenesis protein), gp08 (major capsid protein), gp11 (neck protein), gp13 (major tail protein), gp16 (tape measure protein), gp21, and gp23 (tail fiber proteins) are present in the virion of MED16. Indetermination of potential structural proteins, which were identified by bioinformatics approaches (Table S2) but not detected by proteomics analysis, may be due to the discrepancy of these proteins with sample preparation procedures or/and their low quantity in virions. On the other hand, proteomic analysis led to the experimental identification of endolysin (gp51), conserved hypothetical protein (gp24), and a hypothetical protein with no reliable identity when compared to database entries (gp25) of MED16, suggesting that it may be virion-associated proteins. As seen in Figure 2, all MED16 structural protein-encoding genes are found within a large genome cluster (~21-kb) located just downstream of the packaging module.

### 2.4. Packaging

The packaging machine of tailed bacteriophages usually consists of two essential components: a portal ring and a terminase complex [29]. Most characterized terminases consist of a small subunit (TerS) involved in DNA recognition and a large terminase subunit (TerL) containing the ATPase and the endonuclease activities [30]. The genes associated with MED16 DNA packaging include terminase small subunit (TerS), terminase large subunit (TerL), and portal protein, encoded by ORF01, ORF02, and ORF03, respectively. No conserved domains of MED16 TerS have been identified by BLASTp analysis, whereas HHpred revealed that MED16 TerS (aa 34 to 157) has the best hit to the TerS of Enterobacteria phage P22 (3P9A_G; probability, 99.62%; E-value, 1.8 × 10^−15^). Based on the results of BLASTp analysis, the N-terminus (aa 33 to 278) of MED16 TerL contains the phage_term_2 domain (E-value, 9.99 × 10^−15^) of the Terminase_3 superfamily (cl12054), whereas the C terminus (aa 260 to 448) shows similarity to the Terminase_6C domain (E-value, 3.80 × 10^−6^) of the Terminase_6C superfamily (cl39044). HHpred yielded the best hit of MED16 TerL to the terminase large subunit of deep-sea thermophilic phage D6E (5OE8_A; probability, 100%; E-value, 4.1 × 10^−40^). The portal protein of MED16 contains a conserved DUF4055 domain (E-value, 2.73 × 10^−28^) and yields the best HHpred hit to the portal protein of bacteriophage SPP1 (2JES_M; probability, 99.97%; E-value, 1.6 × 10^−27^).

### 2.5. DNA RRR

The bioinformatics analysis revealed that the genes associated with MED16 DNA replication, recombination, and repair (DNA RRR) include those coding for a single-stranded DNA binding (SSB) protein (ORF27), a recombinase (ORF28), an exodeoxyribonuclease VIII (ORF29), two nucleases (ORF03 and ORF30), a DNA helicase (ORF31) and a DNA primase/replicative helicase (ORF68). However, the genome of MED16 contains no homologues to characterized DNA polymerase genes, suggesting that this phage most likely uses DNA polymerase of the host cell.

The SSB protein (gp27) of MED16 belongs to the RPA_2b-aaRSs_OBF_like superfamily (cl09930), whereas recombinase (gp28) contains an ERF domain (pfam04404) located in its N-terminus. The exodeoxyribonuclease VIII (gp29) contains the C-terminal DUF3799 domain (pfam12684). The gp30 is a VRR-NUC nuclease, whereas gp06 is a putative nuclease which shows a low similarity to a limited number of phage proteins: The best BLASTp hit is a putative homing endonuclease from *Dickeya* phage Kamild (33% identity; E value 5 × 10^−4^). The DNA helicase (gp31) of MED16 belongs to the SSL2 superfamily (cl34083) and DNA primase/replicative helicase (gp68) contains AAA_25 domain (pfam13481) of the P-loop_NTPase superfamily (cl38936). The MED16 genes associated with DNA RRR, with the exceptions of ORF03 and ORF68, are found within the genome cluster (~4.7-kb) located just downstream the structural genes (Figure 2).

### 2.6. Transcription, Translation, Nucleotide Metabolism and DNA Modification

Based on the amino acid sequence similarity, a set of genes encoding products potentially involved in transcription, translation, nucleotide metabolism and DNA modification are present in the genome of MED16. Phage encodes transcriptional regulator (gp26), belonging to the HTH_XRE superfamily (cl22854), while gp41 is a putative DNA-binding protein, which shares similarity to the DNA-binding protein reb1 of *Schizosaccharomyces pombe* (5EYB_A; HHPred probability, 99.42%; E-value, 4.1 × 10^−11^). The reb1 of *Schizosaccharomyces pombe* represents a family of multifunctional proteins that bind to specific terminator sites (Ter) and cause polar termination of transcription catalyzed by RNA polymerase I (pol I) [31]. Thus, it is possible that gp41 of MED16 could also act as a transcription terminator, though further investigation is required to elucidate the function of this protein.

Two genes associated with DNA modification are present in the genome of MED16. The Dam superfamily (cl05442) DNA N-6-adenine-methyltransferase and putative preQ_0_ DNA deoxyribosyltransferase (DpdA) are encoded by ORF32 and ORF38, respectively. The DNA N-6-adenine methyltransferases (Dam MTases) catalyze methyl group transfer from S-adenosyl-L-methionine (AdoMet) to the N6-position of adenine in the DNA polymer and are encoded in genomes of tailed phages containing N6-methyl-2′-deoxyadenine (m6dA) [7]. The N-6-adenine-methyltransferase (gp32) of MED16 shares the highest amino acid similarity (39% identity; E-value 2 × 10^−22^) to N-6-adenine-methyltransferase (gp40) of *Xanthomonas* phage vB_XveM_DIBBI and HHpred yielded the best hit of MED16 gp32 (aa 41 to 206) to the type I restriction enzyme StySJI M protein of *Bacteroides thetaiotaomicron* VPI-5482 (2OKC_B; probability, 96.84%; E-value, 0.018). The preQ_0_ DNA deoxyribosyltransferase of phages and bacteria called DpdAs are homologous to the tRNA-guanine-transglycosylases (TGT in bacteria, arcTGT in archaea)—signature enzymes in the Q and G^+^ tRNA modification pathways that exchange the targeted guanines with 7-deazaguanine precursors [18]. The BLASTp analysis revealed that the closest bacterial homologues of MED16 DpdA are the hypothetical protein (accession CCV45126.1) of *Yersinia enterocolitica* (type O:5,27) str. YE149/02 with 61% identity and E-value 3 × 10^−119^, and the hypothetical protein (accession WP133822589.1) of *Rahnella* sp., with 59% identity and an E-value of 1 × 10^−110^. The closest Med16 DpdA viral homologues (55% identity) are hypothetical proteins gp39 and gp38 of *Salmonella* phages SW9 and SEN1, respectively (Supplementary Table S2 and Figure S3). HHpred yielded the best hit of MED16 DpdA (aa 17 to 199) to the queuine tRNA-ribosyltransferase of *Thermotoga maritima* (2ASH_D; HHPred probability, 99.18%; E-value, 1.0 × 10^−9^).

To identify potentially modified nucleotides in the genome of MED16, and to test the nature of phage DNA modifications, genomic DNA was extracted for LC–MS/MS analysis. Additionally, LC–MS/MS analysis was used to confirm the presence of the potentially modified bases in *Pantoea* phage vB_PagS_Vid5 (Vid5, accession MG948468). It was found that a cluster of genes involved in the biosynthesis of dG^+^ is present in the genome of this phage and the results of restriction analysis indicated the potentially modified genomic DNA (gDNA) [22].

**Figure 3:**
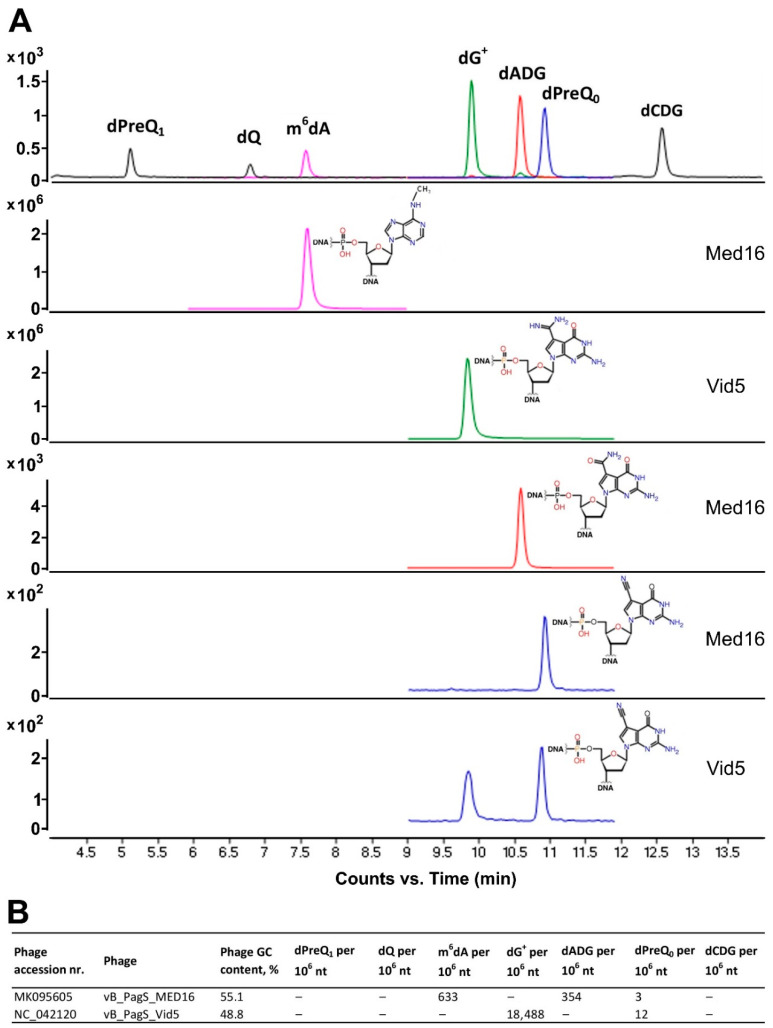
LC–MS/MS chromatograms of digested DNA samples of MED16 and Vid5 phages (**A**) and quantitative results of 7-deazaguanine derivatives and m6dA in MED16 and Vid5 phages (**B**).

LC–MS/MS analysis (Figure 3) revealed that ~7.55% of the Gs in phage Vid5 gDNA are replaced by dG^+^ (18488 modifications per 10^6^ nucleotides,) whereas MED16 gDNA harbors not only m^6^dA (633 modifications per 10^6^ nucleotides, ~0.27% of the Gs), but also dADG (354 modifications per 10^6^ nucleotides, ~0.13% of the Gs). A negligible amount of dPreQ_0_ in the genomes of Vid5 and MED16 (12 modifications per 10^6^ nucleotides, ~0.005% of the Gs and three modifications per 10^6^ nucleotides, ~0.001% of the Gs, respectively) was also detected. Phage Vid5 contains three out of four genes essential for the biosynthesis of preQ_0_ [22], whereas only *dpdA* was detected in the genome of MED16 (Supplementary Table S2). Thus, it is likely that insignificant amounts of the detected dPreQ_0_ in viral genomes could be synthesized by the host enzymes or the exogenous 7-deazapurine precursors could be used.

**Figure 4:**
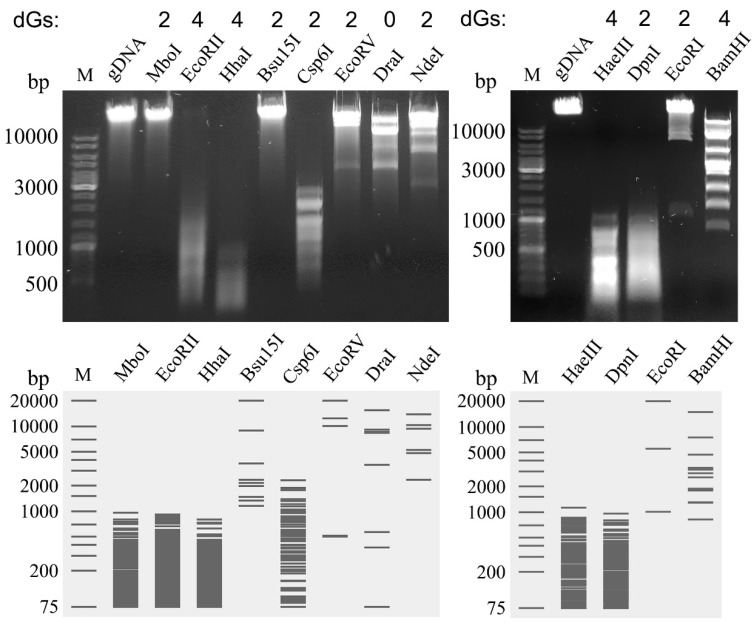
Restriction patterns of phage MED16 genomic DNA by Type II REases. REases are indicated on top of each lane as well as the number of guanine bases (G) in the recognition sequence. The exact number of dADG bases in a particular restriction site is unknown. M—GeneRuler™ DNA Ladder Mix (Thermo Fisher Scientific, Vilnius, Lithuania); gDNA—undigested genomic DNA of MED16. Methylation sensitivity of REases: MboI (Dam methylation-sensitive); EcoRII (Dcm methylation-sensitive); HhaI (CpG methylation-sensitive); Bsu15I (CpG methylation-sensitive, Dam methylation-sensitive); BamHI, Csp6I, DpnI, DraI, EcoRI, EcoRV, HaeIII, NdeI (not Dam methylation-sensitive, not Dcm methylation-sensitive, not CpG methylation-sensitive). Below the gel is the representation of the expected restriction pattern.

In addition, gDNA of MED16 was digested with a set of restriction enzymes that had been shown to be totally or partially inactivated in the presence of various nucleotide modifications [14,18,32]. The results of restriction digestion analysis (Figure 4) revealed that the genomic DNA of phage MED16 was completely resistant to Dam methylation-sensitive REases MboI (↓GATC) and Bsu15I (AT↓CGAT). In contrast, REase with AT recognition sequence, DraI (TTT↓AAA), as well as REases NdeI (CA↓TATG), EcoRII (↓CCWGG), HhaI (GCG↓C), Csp61 (G↓TAC) and BamHI (G↓GATCC), were capable of digesting MED16 DNA despite the presence of guanines in their recognition sequences. Moreover, REases EcoRV (GAT↓ATC), DpnI (GA^m6^↓TC), EcoRI (G↓AATTC) and HaeIII (GG↓CC) also digested phage MED16 DNA despite the presence of GA or GGC sites, which were demonstrated to be specifically modified by DpdA in *Escherichia* phage CAjan [33]. Thus, it is likely that dADG modification, in contrast to m^6^dA, appears to have a minimal inhibitory effect on the activity of REases tested.

**Figure 5:**
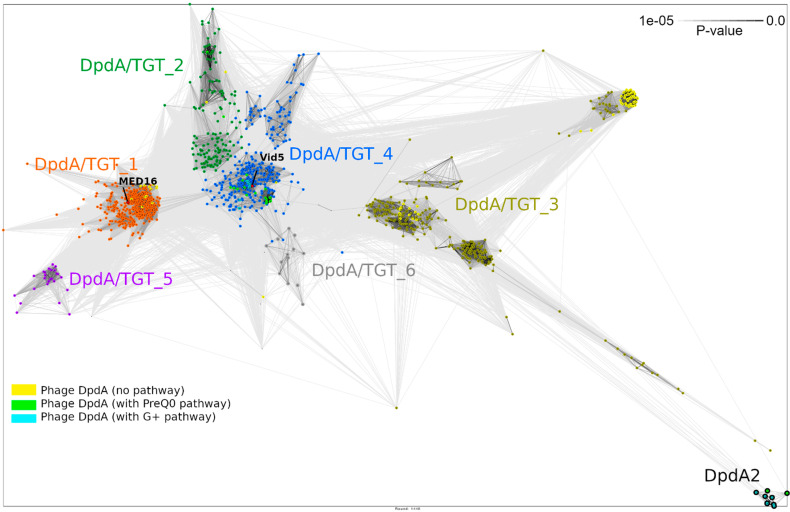
Pairwise comparison of DpdA/TGT proteins. Sequences were clustered and connections are shown at CLANS *p*-value of 1 × 10^−5^.

**Figure 6:**
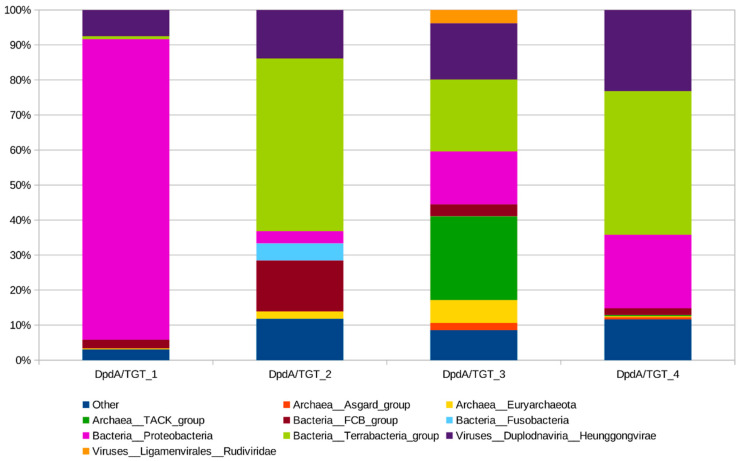
Taxonomic analysis of the largest CLANS groups. Y and X axes show abundance of a specific taxon and largest DpdA/TGT groups, respectively.

To survey the diversity of DpdA-like proteins, we built sequence profiles for MED16 and Vid5 phage DpdA protein homologues. These profiles were used for sensitive searches with HMMER against a database filtered to 90% identity available in MPI Bioinformatics Toolkit. To determine which DpdA-like group MED16 DpdA belongs to, we supplemented full length hits from our searches with characterized DpdA sequences from phages (PMID: 31784519) and clustered the resulting sequence set. Seven clusters were identified after an analysis of networks (Figure 5). DpdA of the MED16 phage belongs to the same cluster (DpdA/TGT_1) as tRNA-guanine-transglycosylase homologues from proteobacteria (Figure 6, Supplementary Table S4) and other phages that encode DpdA but no G^+^ or preQ_0_ biosynthesis protein homologues (Figure 5, PMID: 31784519).

Phylogenetic analysis of DpdA-like proteins corresponds to clustering analysis. CLANS clusters were identified as well-supported branches in a tree (Supplementary Figure S3). A branch of this tree that contains DpdA of MED16 phage have only two viral homologues: gp39 (YP_009883327.1) and gp38 (YP_009217891.1), of the *Salmonella* phages SW9 and SEN1, respectively, which both lack the G^+^ or preQ_0_ biosynthesis protein homologues in their genomes.

### 2.7. Lysis Cassette

The lysis cassette of phage MED16 comprises inner membrane spanin (Rz), outer membrane spanin (Rz1), and endolysin encoded by ORF49, ORF50 and ORF51, respectively. In addition, although gp48 is annotated as MED16-unique protein, which shares no reliable identity to viral proteins and no conserved domains were detected in its structure, based on its length (114 aa), position in the genome and three predicted transmembrane regions, gp48 is a plausible candidate for a type I holin [34]. The endolysin of MED16 shows homology to endolysins from a variety of phages and contains a conserved lyz_endolysin_autolysin domain (cd00737). The MED16 Rz and Rz1 genes have genetic architectures, with the latter being entirely contained within the former in the +1 reading frame (Figure 2). Genes arranged like RzRz1 of MED16 are categorized as two-component spanins (2CS), which have been found to be common in phages of Gram-negative hosts [35].

### 2.8. Phylogenetic Analysis

To determine the phylogenetic relationship between MED16 and its closest relatives, a comparison of the individual genes most often used for the analysis of the evolutionary relationships between bacteriophages [36] was carried out. Therefore, phylogenetic trees based on the alignment of the MED16 major capsid protein, tape measure protein, helicase and terminase large subunit amino acid sequences with those returned by BLASTp homology searches were constructed (Supplementary Figures S4–S7). All phylogenetic trees showed that MED16 has no close relatives among sequenced bacteriophages and represents a distinct branch on the maximum likelihood phylogenetic trees. As seen in Supplementary Figures S4–S7, MED16 seems to occupy a somewhat intermediate position between siphophages belonging to the genera *Eiauvirus*, *Murrayvirus*, *Tlsvirus* and *Webervirus*.

**Figure 7:**
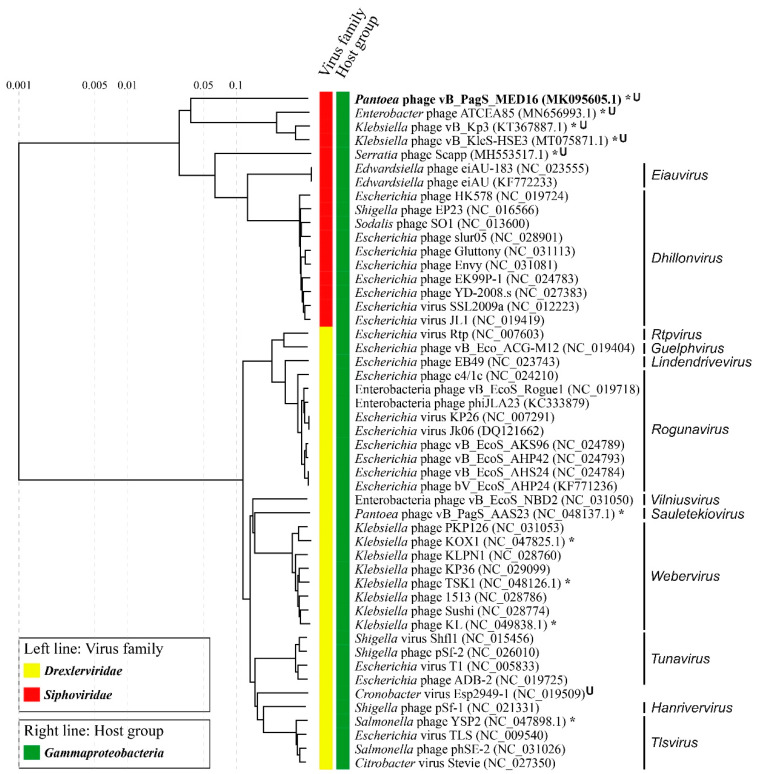
ViPTree generated proteomic tree of *Pantoea* phage vB_PagS_MED16 and dsDNA viruses represented in the rectangular view. The tree was constructed by BIONJ based on genomic distance matrixes and mid-point rooted. Branch lengths are logarithmically scaled from the root of the entire proteomic tree. The numbers at the top represent the log-scaled branch lengths based on the SG (normalized tBLASTx scores) values.

To obtain a more detailed picture of the phylogenetic relationships of MED16 and its closest relatives, the overall nucleotide sequence identity was calculated using PASC, and a comparative total proteome comparison was performed using ViPTree web-service. Notably, since the virus–host database used by the ViPTree does not contain the genome sequences of a number of phages that share phylogenetic relatedness with MED16, those were added to the query along with the genome of MED16. Based on the whole-proteome alignment of MED16 and its closest relatives, it was demonstrated that the phage is related to unclassified members of the family *Siphoviridae*: The *Klebsiella* phages vB_KleS-HSE3, vB_Kp3 and the *Enterobacter* phage ATCEA85 (Figure 7). However, the phylogenetic relationship between MED16 and all the aforementioned phages is distant, suggesting that phage MED16 has an evolutionarily but not yet identified lineage within the siphoviruses.

**Figure 8:**
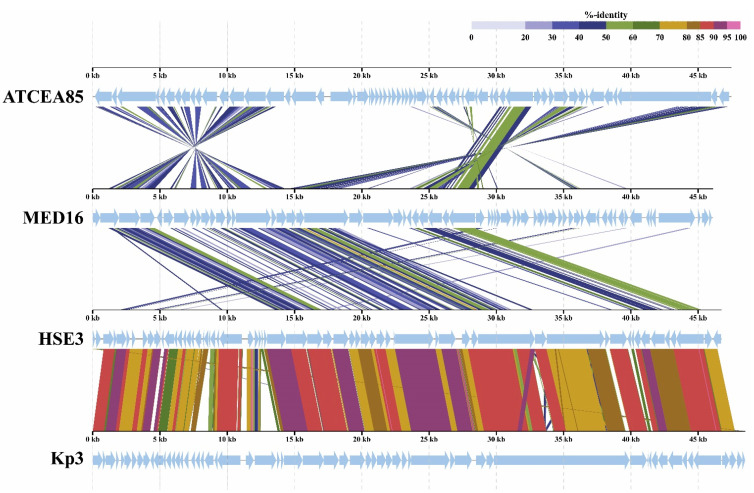
ViPTree generated whole-proteome alignment of *Pantoea* phage vB_PagS_MED16 and its closest relatives: *Klebsiella* phages vB_KleS-HSE3, Kp3 and *Enterobacter* phage ATCEA85. Colored lines in the alignment indicate tBLASTx results (E-value < 1 × 10^−2^). Positions of each sequence were automatically adjusted (i.e., circularly permuted and reverse stranded) for a clear representation of collinearity between genomes.

To determine the most homologous regions in the genomes of MED16 and its closest relatives, which are the *Klebsiella* phages vB_KleS-HSE3, vB_Kp3 and *Enterobacter* phage ATCEA85, genome alignment was performed by using ViPTree. Genomes of all bacteriophages share several regions of nucleotide similarity that cover the essential structural and virion morphogenesis protein-encoding genes, as well as genes related to DNA metabolism and modification (Figure 8). Nevertheless, the nucleotide-based virus overall nucleotide sequence identity between MED16 and its closest relatives are quite low and ranges from 25.98% (MED16 vs. *Klebsiella* phage vB_KleS-HSE3) to 22.70% (MED16 vs. *Escherichia* phage HK578) (Supplementary Table S5).

According to the Bacterial and Archaeal Viruses Subcommittee (BAVS) of the ICTV, a genus is described as a cohesive group of viruses sharing a high degree (>70%) of nucleotide identity of the full genome length [37]. Following this, and based on the results of the comparative genome sequence analysis performed during this study, we consider that bacteriophage MED16 cannot be classified to any genus currently recognized by ICTV and likely represents a new one within the siphoviruses.

## 3. Discussion

In this study, we present a detailed analysis of the bacteriophage MED16, which was originally isolated from the outwash of thicket shadbush collected in Lithuania using a local isolate, *Pantoea agglomerans* strain BSL, as the host for phage propagation. *Pantoea* is a genus of Gram-negative, non-sporulating, yellow-pigmented, and highly diverse bacteria of the order *Enterobacterales*. Although members of this genus have been found to predominate in the phyllosphere of various plants, both as endophytes and epiphytes, *Pantoea* have been isolated from many soil, water, and nosocomial environments [27,38,39]. Some strains of *P. agglomerans* have been used for a variety of applications including biological control against phytopathogenic bacteria and fungi, bioremediation, and therapeutics [40,41,42]. On the other hand, *Pantoea* isolates that are opportunistic pathogens of plants, animals and humans have been reported as well [43]. Despite the importance of bacteriophages in ecology, evolution and shaping the general biology of bacteria, and their promising use as biocontrol tools against pathogenic bacteria [44,45,46], *Pantoea*-infecting viruses still remain poorly understood.

Only 14 bacteriophages with completely sequenced genomes, annotated as *Pantoea* phages, have been published or deposited in Genbank to date (Supplementary Table S6) [22,32,47,48,49,50]. Nevertheless, it was demonstrated that broad host range bacteriophages, including a number of *Erwinia* phages [51,52,53,54,55,56,57,58] and *Serratia* phage phiOT8 [59], were also able to infect bacteria from the genus *Pantoea*. This is not surprising given that *Pantoea* and *Erwinia* form a monophyletic group within the family *Erwiniaceae* [27,60], and some broad-host range bacteriophages are active in phylogenetically closely related bacterial species [61]. However, to our knowledge, none of the guanosine hypermodifications have been experimentally validated in the genomes of *Pantoea*-infecting bacteriophages. Thus, the data presented in this study not only provide novel insights into bacteriophages from phyllospheres, but also expand our understanding of newly discovered hypermodifications in the genomes of bacterial viruses. On the other hand, a number of research questions, related to the presence of dADG in the genome of MED16, still remain unanswered, including: (i) What evolutionary benefit, if any, might this modification provide to the phage? (ii) What are the mechanisms of biosynthesis and replacement of dG with dADG? (iii) What is the phylogenetic relationship between DpdA and its homologues and other viruses and microorganisms?

As mentioned previously, a pivotal function of modifications in phage genomes is thought to be prevention of cleavage by host restriction endonucleases [4,5,6,7]. Indeed, bacteriophage gDNAs containing modified nucleotides are partially or completely resistant to cleavage by a variety of REases in vitro. However, although relatively similar amounts of non-canonical nucleotides, m^6^dA and dADG (~0.27% of the As and ~0.13% of the Gs, respectively), were detected in the gDNA of MED16, the results of restriction digestion analysis suggest that dADG, in contrast to m^6^dA, has no noticeable effect on the activity of REases tested (Figure 4). In comparison, it was demonstrated that 100% of the dG residues are replaced by dADG in genomic DNA of *Campylobacter* phages, CP220 and CPt10 [19], whereas Halovirus HVTV-1, *Vibrio* phage nt-1, *Salmonella* phage 7–11 and *Mycobacterium* phage Rosebush contain relatively small or even negligible amounts of dADG (~0.05%, ~0.035%, ~0.02% and ~0.003% of the Gs, respectively) [18]. However, potentially regarding other modifications detected in the genomes of aforementioned phages or due to the lack of experimental evidence, the exact effect of dADG on the activity of REases has still not precisely been determined. For example, Halovirus HVTV-1 gDNA was found to resist restriction by all enzymes tested, but it contains not only dADG, but also dPreQ_1_ and dG+ (~30.00% and ~0.008% of the Gs, respectively) in its genome [18].

Nevertheless, it is still an open question as to whether dG replacement with dADG (albeit on a small scale) in the gDNA of MED16 has any meaning to phage or its host. The DNA modifications in phages have been proposed to have a number of other functions such as inhibition of some but not all CRISPR–Cas systems, as demonstrated in *Escherichia* phage T4 [62,63]; playing a pivotal role in phage gene expression, as shown in *Escherichia* phage Mu [64] and *Bacillus* phage SPO1 [65]; initiation of DNA packaging into the viral capsid, as indicated in *Escherichia* phage P1 [66]; and maintenance of the stability of DNA when compactly packed within a viral capsid, as detected in the *Delftia* phage ΦW-14 [67]. Whether dADG, present in gDNA of MED16, is related to some of the functions mentioned remains unclear, though more detailed studies are needed to elucidate the importance of this hypermodification in genome of the MED16 phage.

However, the precise mechanism by which dG is replaced by dADG in gDNA of bacteriophages remains poorly explained. The proposed biosynthesis pathways for the 2′-deoxy-7-deazaguanine modifications in DNA of bacteriophages and bacteria suggest that dADG is synthesized from the precursor base 7-cyano-deazaguanine (PreQ_0_) [13,18,68]. In both archaea and bacteria, preQ_0_ is generated from GTP in a pathway that has been fully characterized and includes four preQ_0_ synthesis genes (*folE*, *queD*, *queE*, *queC*). However, an in silico search for phage genomes that could harbor 7-deazaguanine derivatives in their genomic DNA revealed a group of 76 phages deposited in GenBank, which were found to encode DpdA but no G^+^ or preQ_0_ biosynthesis protein homologues [18]. The mass spectrometric analysis of genomic DNAs of two representatives of this group, *Salmonella* phage 7–11 (NC_015938) and *Mycobacterium* phage Orion (DQ398046), demonstrated that phage 7–11 was unexpectedly modified by dADG, whereas phage Orion lacked any 7-deazaguanine modifications in its DNA, suggesting the host-dependent preQ_0_ synthesis pathway (the host of phage Orion, *Mycobacterium smegmatis*, encodes no genes for the preQ_0_ biosynthesis) [18]. Although there are no data about the preQ_0_ biosynthesis genes potentially encoded in the host of MED16 (the genome of *Pantoae agglomerans* strain BSL is unsequenced to date), but all four preQ_0_ synthesis genes (*folE*, *queD*, *queE*, and *queC*) were detected in a number of bacteria from the genus of *Pantoea,* including *Pantoea agglomerans* 299R (1261128.3) and *Pantoea agglomerans* IG1 (1110694.4) [18], which suggests that the host of MED16 is most likely to also have these genes. On the other hand, we cannot exclude the possibility that preQ_0_ also may be uptaken from the environment as a secondary metabolite, secondary metabolite precursor [69,70] or tRNA degradation product [71].

Additionally, it is still unknown whether dADG of MED16 is synthesized and incorporated into DNA during replication or if is it modified after replication. In archaea, preQ_0_ is directly incorporated into tRNA by arcTGT before being further modified by the amidotransferases ArcS, GatQueC, or QueF-L [72,73,74]. In bacteria, preQ_0_ is reduced to 7-aminomethyl-7-deazaguanine (preQ_1_) by QueF [75] before TGT incorporates it in tRNA [76], where it is further modified to Q in two steps [77,78,79]. Hutinet and colleagues [18] proposed that, in bacteriophages, preQ_0_ is directly inserted into gDNA by DpdA/DpdA2, resulting in production of dPreQ_0_ (phages Cajan, Rosebush and nt-1), which may be further modified by ArcS, Gat-QueC, or QueF-L to dPreQ_1_ (phages HVTV-1 and Dp-1), dG^+^ (phages 9g, HVTV-1 and nt-1), and dADG (phages HVTV-1, Rosebush, 7-11 and nt-1). However, most of the final steps of proposed pathways await experimental elucidation.

Finally, the evolutionary origin of phages’ DpdA and the phylogenetic relationships of these proteins and their homologues with other viruses and microorganisms also remain unknown. Phylogenetic analysis of MED16 DpdA and its closest homologues corresponds to clustering analysis (Figure 5 and Figure 6, Supplementary Figure S3). These results indicate that DpdA of MED16 has a closer phylogenetic relationship with bacterial tRNA-guanine-transglycosylase homologues than with viral proteins and suggest its bacterial origin. However, further in-depth research is needed to confirm this hypothesis.

## 4. Materials and Methods

### 4.1. Phages and Bacterial Strains

Bacteriophage MED16 was originally isolated from the outwash of thicket shadbush collected in Lithuania. *Pantoea agglomerans* strain BSL was used as the host for MED16 isolation, propagation and phage growth experiments. The bacterial strains used in this study for host range determination of MED16 are listed in Table S1. For all of the phage experiments, the bacteria were cultivated in Luria–Bertani broth (LB) or LB agar.

### 4.2. Phage Isolation, Propagation and Purification Techniques

Phage isolation was performed by using the enrichment of phages in the source material technique as described previously [22]. Phage titration was performed by using the soft agar overlay method [80], with minor modifications. Briefly, 0.1 mL of diluted phage suspension was mixed with 0.5 mL of indicator cells (OD_600_–0.5). The mixture then was added to 2.5 mL of 0.4% (*w*/*v*) soft agar and poured over the 1.2% LB agar plate as a uniform layer. The plates were incubated for 1–10 days at 3–40 °C before the enumeration of plaques. Bacteriophage was purified by performing five consecutive transfers of phages from individual plaques to new bacterial cell lawns. Phage was propagated from a single plaque suspension that was added to the exponentially growing liquid culture of *P. agglomerans* strain BSL. The culture then was incubated at 27 °C with a mild shaking until the complete lysis of the cells (~3–4 h). The cell debris was removed from the lysate by centrifugation at 5000× *g* for 15 min at 4 °C. The supernatant was collected and centrifuged at 16,000× *g* for 1 h at 4 °C. The resulting pellet was soaked in 1 mL of SM buffer (100 mM NaCl, 8 mM MgSO4, 50 mM Tris-HCl, pH 7.5) containing 0.1 mL CHCl_3_ and DNase I (2 units per mL; Thermo Fisher Scientific, Vilnius, Lithuania) at 4 °C overnight. Next day, the bacterial debris was removed by centrifugation at 5000× *g* for 10 min at room temperature. The supernatant was then subjected to further purification by CsCl step gradient [81], as described by Šimoliūnas et al. [82]. The adsorption tests and efficiency of plating (EOP) experiments were carried out as described by Kropinski [83] and Žukauskienė et al. [32], respectively.

### 4.3. Transmission Electron Microscopy

The CsCl density gradient-purified phage particles were diluted to approximately 10^11^ PFU/mL with distilled water, and 10 µL of the sample was directly applied on the carbon-coated nickel grid (Agar Scientific, Essex, UK). After 1 min, the excess liquid was drained with filter paper and stained with two successive drops of 2% uranyl acetate (pH 4.5) for 1 min. The sample then was dried and examined using a Tecnai G2 F20 X-TWIN transmission electron microscope (FEI, Hillsboro, OR, USA).

### 4.4. DNA Isolation and Restriction Analysis

The aliquots of high-titer (10^11^–10^12^ PFU/mL) phage suspension were subjected to phenol/chloroform extraction and ethanol precipitation, as described by Carlson and Miller [84]. The isolated phage DNA was subsequently used in restriction analysis, for PCR, or it was subjected to genome sequencing. The restriction digestion was performed with Bsu15I, Csp6I, DraI, EcoRII, EcoRV, HhaI, MboI, and NdeI restriction endonucleases (Thermo Fisher Scientific, Vilnius, Lithuania), according to the supplier’s recommendations. The DNA fragments were separated by electrophoresis in a 0.8% agarose gel containing ethidium bromide. The restriction analysis was performed in triplicate and Figure 4 shows a representative result.

### 4.5. Genome Sequencing and Analysis

The complete genome sequence of MED16 was determined using Illumina DNA sequencing technology at BaseClear (Leiden, The Netherlands). DNA libraries were prepared using Illumina Nextera XT library preparation. The 150 bp length paired-end sequence reads were generated using the Illumina NovaSeq 6000.

FASTQ read sequence files were generated using bcl2fastq version 2.20 (Illumina). Initial quality assessment was based on data passing the Illumina Chastity filtering. Subsequently, reads were mapped to the PhiX genome using bowtie2 version 2.2.6. Aligned reads were removed from the fastq file. Reads containing (partial) adapters were clipped (up to a minimum read length of 50 bp). The fastq-mcf tool from the ea-utils package version 1.04 was used to trim remaining adapter sequences. The second quality assessment was based on the remaining reads using the FASTQC quality control tool version 0.11.5 available online: https://www.bioinformatics.babraham.ac.uk/projects/fastqc/ (accessed on 3 June 2021). The quality of Illumina reads was improved using the error correction tool BayesHammer [85] as bundled with SPAdes packages version 3.10.1 [86], which was used to assemble error-corrected reads into contigs. The order of contigs, and the distances between them, were estimated using the insert size information derived from an alignment of the paired-end reads to the draft assembly. Therefore, contigs were linked together and placed into scaffolds using SSPACE version 2.3 [87]. Using Illumina reads, gapped regions within scaffolds were (partially) closed using GapFiller version 1.10 [88]. The assembly errors and the nucleotide disagreements between the Illumina reads and scaffold sequences were corrected using Pilon version 1.21 [89]. Finally, the reads of MED16 were assembled into a single linear contig of 46,180 bp (130,345 mapped reads; 387.06 average coverage). The ends of the assembled contig were confirmed using PCR, followed by Sanger sequencing reactions at Macrogen (Seoul, South Korea). A PCR fragment was obtained by the amplification of MED16 phage wild-type DNA using MED16_F1 5′-AGCCTGTCGTTGTCAATCTC-3′ and MED16_R1 5′-TGTCGGGAGTTCTCAAAGAAC-3′ primers. No defined genomic termini were identified, and to preserve gene contiguity, the genome start point was selected from the predicted terminase small subunit gene.

The open reading frames (ORFs) were predicted with Geneious Prime 2020 (http://www.geneious.com (accessed on 3 June 2021)), using a minimum ORF size of 60 nt. The analysis of the genome sequences was performed using psiBLAST, BLASTp, Fasta-Protein, Fasta-Nucleotide, Transeq available online: http://www.ebi.ac.uk/Tools/st/emboss_transeq (accessed on 3 June 2021), Clustal Omega available online: http://www.ebi.ac.uk/Tools/msa/clustalo (accessed on 3 June 2021), and DNA sequence editor available online: http://www.biocourseware.com/iphone/dnaseqeditor/index.htm (accessed on 3 June 2021), as well as HHPred, HHblits, HMMER, and HHsenser [90,91]. The tRNAscan-SE 1.21 available online: http://lowelab.ucsc.edu/tRNAscan-SE (accessed on 3 June 2021) and ARAGORN available online: http://130.235.244.92/ARAGORN (accessed on 3 June 2021) were used to search for tRNAs. Maximum likelihood phylogenetic trees were built with an IQ-Tree using Ultrafast Bootstrap Approximation branch support [92] (options “-alrt 2000 -bb 2000 -nm 2000 -nt 80”). ViPTree was used for the total proteome comparisons [93]. The overall nucleotide sequence identity was calculated using PASC [94].

### 4.6. Analysis of Structural Proteins

An analysis of the structural proteins of MED16 virions was performed using a modified filter-aided sample preparation (FASP) protocol, followed by LC–MS/MS analysis, as described previously [95].

### 4.7. LC–MS/MS Analysis of Phage DNA Modifications

DNA analysis was performed as described previously [13], with some modifications. Purified DNA (10 μg) was hydrolyzed in 10 mM Tris-HCl (pH 7.9), with 1 mM MgCl_2_ with Benzonase (20U), DNase I (4U), calf intestine phosphatase (17U) and phosphodiesterase (0.2U) for 16 h at ambient temperature. Following passage through a 10 kDa filter to remove proteins, the filtrate was analyzed by liquid chromatography-coupled triple quadrupole mass spectrometry (LC–MS/MS).

Quantification of the modified 2′-deoxynucleosides (dADG, dCDG, dQ, dPreQ0, dPreQ1, dG+ and m6dA) and the four canonical deoxyribonucleosides (dA, dT, dG, and dC) was achieved by LC–MS/MS and liquid chromatography-coupled diode array detector (LC–DAD), respectively. Aliquots of hydrolyzed DNA were injected onto a Phenomenex Luna Omega Polar C18 column (2.1 × 100 mm, 1.6 μm particle size) equilibrated with 98% solvent A (0.1% *v*/*v* formic acid in water) and 2% solvent B (0.1% *v*/*v* formic acid in acetonitrile) at a flow rate of 0.25 mL/min and eluted with the following solvent gradient: 12% B for 10 min, 1 min ramp to 100% B for 10 min, 1 min ramp to 2% B for 10 min. The HPLC column was coupled to an Agilent 1290 Infinity DAD and an Agilent 6490 triple quadruple mass spectrometer (Agilent, Santa Clara, CA, USA). The column was kept at 40 °C and the auto-sampler was cooled at 4 °C. The UV wavelength of the DAD was set at 260 nm and the electrospray ionization of the mass spectrometer was performed in positive ion mode with the following source parameters: drying gas temperature—200 °C, with a flow of 14 L/min, nebulizer gas pressure—30 psi, sheath gas temperature—400 °C with a flow of 11 L/min, capillary voltage—3000 V and nozzle voltage—800 V. Compounds were quantified in multiple reaction monitoring (MRM) mode with the following transitions: *m*/*z* 310.1 194.1, 310.1 177.1, 310.1 293.1 for dADG; *m*/*z* 311.1 177.1, 311.1 79, for dCDG; *m*/*z* 394.1 163.1, 394.1 146.1, 394.1 121.1 for dQ; *m*/*z* 292.1 176.1, 176.1 159.1, 176.1 52.1 for dPreQ0; *m*/*z* 296.1 163.1, 296.1 121.1, 296.1 279.1 for dPreQ1; *m*/*z* 309.1 193.1, 309.1 176.1, 309.1 159.1 for dG+; *m*/*z* and 266.1 150.1, 266.1 108.1, 266.1 55.1 for m6dA. External calibration curves were used for the quantification of the modified 2′-deoxynucleosides and the four canonical deoxyribonucleosides. The calibration curves were constructed from replicate measurements of eight concentrations of each standard. A linear regression with r2 > 0.995 was obtained in all relevant ranges. The limit of detection (LOD), defined by a signal-to-noise ratio (S/N) of 3, ranged from 0.1 to 1 fmol for the modified 2′-deoxynucleosides. Data acquisition and processing were performed using MassHunter software (Agilent, Santa Clara, CA, USA).

### 4.8. Bioinformatic Analysis of DpdA-Like Proteins

Homologues of DpdA proteins from phages MED16 and Vid5 were found using a BLASTp search. DpdA homologues belonging to phages were extracted from the results. DpdA-like sequences were aligned using MAFFT (option L-INS-i) [96]. Multiple sequence alignments were used as queries for HMMER searches against the nr90_15_Jan database in MPI Bioinformatics Toolkit [97]. Full length sequences of hits were extracted and supplemented with phage DpdA sequences from [18]. The resulting set of sequences was clustered with CLANS [98]. To prepare DpdA-like protein set for phylogenetic analysis, sequences lacking connections at *p*-value 1 × 10^−5^ and highly diverged DpdA2 group were removed. Remaining sequences were filtered to 90% identity with CD-HIT version 4.6 [99] (options “-d 0 -c 0.9 -T 0”) and aligned with MAFFT. TrimAl [100], with a gap threshold of 0.05 (option “-gt 0.05”), was used to trim the alignment. A maximum likelihood phylogenetic tree, whose fragment is shown in Figure S3, was built with an IQ-Tree using Ultrafast Bootstrap Approximation branch support [92] (options “-alrt 2000 -bb 2000 -nm 2000 -nt 80”). ModelFinder selected the “LG+R10” model of evolution as the best fit for our alignment.

### 4.9. Nucleotide Sequence Accession Numbers

The complete genome sequence of the *Pantoea* bacteriophage MED16 was deposited in the EMBL nucleotide sequence database under accession number MK095605. The raw sequence reads are available in the SRA database under accession number SRX11233249 (BioProject number PRJNA741396 and BioSample number SAMN19872044).

## 5. Conclusions

In conclusion, we have shown that the *Pantoea* sp.-infecting bacteriophage MED16 is a siphovirus possessing the *dpdA* gene and dADG-modified DNA, which has never been experimentally validated in the genomes of *Pantoea* bacteriophages. In addition, our results indicate that MED16 is substantially distinct from all of the previously described phages and may be considered as a representative of a novel genus within the siphoviruses.

## Data Availability

The complete genome sequence of MED16 is available in the EMBL nucleotide sequence database under accession number MK095605.The raw sequence reads of MED16 are available in the SRA database under accession number SRX11233249 (BioProject number PRJNA741396 and BioSample number SAMN19872044).

## Supplementary Materials

The following are available online at https://www.mdpi.com/article/10.3390/ijms22147333/s1.
