# Supplementary material for: Pantoea Bacteriophage vB_PagS_MED16—A Siphovirus Containing a 2′-Deoxy-7-amido-7-deazaguanosine-Modified DNA
Source: Int J Mol Sci. 2021 Jul 8;22(14):7333. doi: 10.3390/ijms22147333 (PMC8306585; doi:10.3390/ijms22147333)
Supplement: Supplementary file 1 [file ijms-22-07333-s001.zip › Supplementary material/IJMC Supplementary material.pdf]

*Supplementary material*

# *Pantoea* bacteriophage vB_PagS_MED16 – a siphovirus containing a 2′-deoxy-7-amido-7-deazaguanosine-modified DNA

**Monika Šimoliūnienė[1], Emilija Žukauskienė[1], Lidija Truncaitė[1], Liang Cui[2], Geoffrey Hutinet[3], Darius Kazlauskas[4], Algirdas Kaupinis[5], Martynas Skapas[6], Valérie de Crécy-Lagard[3,7], Peter C. Dedon [2,8], Mindaugas Valius[5], Rolandas Meškys[1] and Eugenijus Šimoliūnas[1,*]**

1 Department of Molecular Microbiology and Biotechnology, Institute of Biochemistry, Life Sciences Centre, Vilnius University, Saulėtekio av. 7, LT-10257 Vilnius, Lithuania; monika.simoliuniene@gmc.vu.lt (M.Š.); emilija.zukauskiene@bchi.stud.vu.lt (E.Ž.); lidija.truncaite@bchi.vu.lt (L.T.); rolandas.meskys@bchi.vu.lt (R.M.);
2 Singapore-MIT Alliance for Research and Technology, Antimicrobial Resistance Interdisciplinary Research Group, Campus for Research Excellence and Technological Enterprise, Singapore 138602, Singapore; liangcui@smart.mit.edu (L.C.); pcdedon@mit.edu (P.C.D.);
3 Department of Microbiology and Cell Science, University of Florida, Gainesville, FL 32611, USA; ghutinet@ufl.edu (G.H.); vcrecy@ufl.edu (V.C.L.);
4 Department of Bioinformatics, Institute of Biotechnology, Life Sciences Centre, Vilnius University, Saulėtekio av. 7, LT-10257 Vilnius, Lithuania; darius.kazlauskas@bti.vu.lt (D.K.);
5 Proteomics Centre, Institute of Biochemistry, Life Sciences Centre, Vilnius University, Saulėtekio av. 7, LT-10257 Vilnius, Lithuania; algirdas.kaupinis@gf.vu.lt (A.K.); mindaugas.valius@bchi.vu.lt (M.V.)
6 Center for Physical Sciences and Technology, Saulėtekio av. 3, LT-10257 Vilnius, Lithuania; martynas.skapas@gmail.com
7 University of Florida, Genetics Institute, Gainesville, Florida 32610, USA; vcrecy@ufl.edu (V.C.L.);
8 Department of Biological Engineering and Center for Environmental Health Sciences, Massachusetts Institute of Technology, Cambridge, MA 02139, USA; pcdedon@mit.edu (P.C.D.);
* Correspondence: eugenijus.simoliunas@bchi.vu.lt (E.Š.); Tel.: +3706-507-0467

**Table S1.** Bacterial strains used in this study to determine the host range of phage MED16.

| Strain | Relevant characteristics | Source or reference |
|---|---|---|
| *Acinetobacter baumannii* #46 | | Prof. E. Sužiedėlienė |
| *Citrobacter freundii* | | Prof. E. Sužiedėlienė |
| *Erwinia carotovora* 8982 | | Prof. E. Sužiedėlienė |
| *Erwinia carotovora* 961–63 | | Prof. E. Sužiedėlienė |
| *Erwinia piriflorinigrans* 26166 | type strain | DSMZ |
| *Erwinia billingiae* 17872 | type strain | DSMZ |
| *Escherichia coli* B40 | *sup*D | Dr. L. W. Black |
| *Escherichia coli* $B^E$ | $sup^0$ | Dr. L. W. Black |
| *Escherichia coli* BL21 | $F^–$ *dcm ompT hsdS* ($rB^–mB^–$) *gal* | Novagen |
| *Escherichia coli* BW25113 | [Δ(*araD–araB*)*567* Δ(*rhaD–rhaB*)*568 ΔlacZ4787* (::rrnB-3) *hsdR514 rph-1*] | [1] |
| *Escherichia coli* DH10β | $F^–$ *endA1 recA1 galE15 galK16 nupG rpsL* Δ*lacX74* Φ80*lacZ*ΔM15 *araD139* Δ(*ara,leu*)7697 *mcrA* Δ(*mrr-hsdRMS-mcrBC*) $\lambda^-$ | Invitrogen |
| *Escherichia coli* MG1655 | $F^-$ $lambda^-$ *ilvG*$^-$ *rfb*-50 *rph*-1 | Prof. E. Sužiedėlienė |
| *Escherichia coli* MH1 | *araD139* Δ*lacX74 galU galK hsr hsm rpsL* | Dr. K. N. Kreuzer |
| *Klebsiella* sp. KV-3 | Veterinary isolate, $Amp^r$, $Str^r$, $Tet^r$, $Kan^s$, $Gm^s$, $Nc^s$,$Cl^{r/s}$ | [2] |
| *Pantoea agglomerans* ARC | environmental isolate | [3] |
| *Pantoea agglomerans* AUR | environmental isolate | [3] |
| ***Pantoea agglomerans* BSL** | environmental isolate | [3] |
| *Pantoea agglomerans* DDM | environmental isolate | [3] |
| *Pantoea agglomerans* MMG | environmental isolate | [3] |
| *Pantoea agglomerans* SER | environmental isolate | [3] |
| *Pantoea agglomerans* 3493 | type strain | DSMZ |
| *Pantoea conspicua* 24241 | type strain | DSMZ |
| *Pseudomonas aeruginosa* PAO1 | | Prof. E. Sužiedėlienė |
| *Salmonella enterica* ser. Typhimurium 292 | | Prof. E. Sužiedėlienė |

MED16-sensitive strain is in bold.

**Table S2.** MED16 ORFs-having homologues with reliable identity (E-values >0.001) in other viruses or cellular organisms.

| MED16 ORF (position) | Protein_id predicted function (protein length aa) | Significant match (protein length aa) | Identity aa %/ similarity aa% (length of the overlapping segment) | E value |
|---|---|---|---|---|
| ORF01 (1..567) | AZS06241.1 terminase small subunit (188) | ALJ98181.1 hypothetical protein *Klebsiella* phage vB_Kp3 (171) | 31/44 (150) | 6e-12 |
| ORF02 (560..1957) | AZS06242.1 terminase large subunit (465) | ADV36428.1 phage terminase large subunit *Edwardsiella* phage eiAU (460) | 54/69 (451) | 8e-163 |
| ORF03 (1969..3516) | AZS06243.1 portal protein (515) | YP_006990387.1 phage structural protein *Escherichia* virus JL1 (506) | 58/70 (486) | 0.0 |
| ORF04 (3509..4615) | AZS06244.1 head morphogenesis protein (368) | YP_006590036.1 hypothetical protein B887_gp31 *Cronobacter* phage phiES15 (367) | 46/51 (309) | 1e-61 |
| ORF05 complement (4588..4764) | AZS06245.1 hypothetical protein (58) | no significant match | | |
| ORF06 complement (4764..5162) | AZS06246.1 putative endonuclease (132) | AYN55760.1 putative homing endonuclease *Dickeya phage Kamild* (294) | 33/45 (124) | 5e-04 |
| ORF07 (5281..5985) | AZS06247.1 putative scaffolding protein (234) | YP_002720075.1 hypothetical protein AV954_gp09 *Escherichia* virus SSL2009a (233) | 49/65 (201) | 4e-43 |
| ORF08 (6046..7176) | AZS06248.1 major capsid protein (376) | YP_006590038.1 putative major capsid protein *Cronobacter* phage phiES15 (320) | 43/61 (285) | 2e-64 |
| ORF09 (7263..7709) | AZS06249.1 putative head-tail adaptor (148) | YP_002720072.1 hypothetical protein AV954_gp11 *Escherichia* virus SSL2009a (176) | 39/53 (167) | 2e-31 |
| ORF10 (7712..8068) | AZS06250.1 head completion protein (118) | YP_009152271.1 putative structural protein *Escherichia* phage YD-2008.s (117) | 46/56 (118) | 7e-29 |
| ORF11 (8068..8733) | AZS06251.1 putative neck protein (221) | ALJ98192.1 hypothetical protein *Klebsiella* phage vB_Kp3 (227) | 36/52 (203) | 5e-36 |
| ORF12 (8730..9137) | AZS06252.1 putative tail completion protein (135) | YP_009004697.1 hypothetical protein O197_50 *Edwardsiella* phage eiAU-183 | 41/45 (134) | 3e-24 |
| ORF13 (9162..9875) | AZS06253.1 major tail protein (237) | YP_009004698.1putative major tail protein *Edwardsiella* phage eiAU-183 (239) | 62/65 (237) | 3e-93 |
| ORF14 (9966..10337) | AZS06254.1 tail assembly chaperone (123) | YP_004934044.1 hypothetical protein HK639_14 *Escherichia* phage HK639 (112) | 45/47 (111) | 2e-26 |
| ORF15 (10361..10648) | AZS06255.1 hypothetical protein (95) | YP_009004700.1 hypothetical protein O197_53 *Edwardsiella* phage eiAU-183 (108) | 55/64 (102) | 2e-26 |
| ORF16 (10654..13242) | AZS06256.1 tape measure protein (862) | YP_004934045.1 tail length tape measure protein *Escherichia* phage HK639 (1024) | 37/40 (850) | 2e-101 |
| ORF17 (13294..13656) | AZS06257.1 minor tail protein (121) | YP_004934049.1 minor tail protein *Escherichia* phage HK639 (135) | 50/55 (106) | 1e-16 |
| ORF18 (13653..14399) | AZS06258.1 minor tail protein (248) | YP_009004650.1 minor tail protein l *Edwardsiella* phage eiAU-183 (256) | 56/62 (257) | 7e-94 |
| ORF19 (14409..15143) | AZS06259.1 putative tail-associated protein (244) | YP_004934051.1 tail assembly protein K Escherichia phage HK639 (243) | 68/74 (241) | 6e-119 |
| ORF20 (15134..15733) | AZS06260.1 tail assembly protein (199) | YP_009004652.1 putative tail assembly protein *Edwardsiella* phage eiAU-183 (198) | 64/80 (192) | 9e-89 |
| ORF21 (15733..19008) | AZS06261.1 tail fiber protein (1091) | YP_004934053.1 host specificity protein *Escherichia* phage HK639 (1154) | 52/58 (1177) | 0.0 |

| | | | | |
|---|---|---|---|---|
| ORF22 (19001..20095) | AZS06262.1 hypothetical protein (364) | NP_892069.1 hypothetical protein PY54p23 *Yersinia* phage PY54 (340) | 28/42 (370) | 3e-24 |
| ORF23 (20106..22346) | AZS06263.1 putative tail fiber protein (746) | ARB10877.1 minor tail protein *Salmonella* phage 29485 (432) | 44/48 (147) | 2e-28 |
| ORF24 (22346..23068) | AZS06264.1 structural protein (240) | QAX92300.1 hypothetical protein LIET2_gp048 *Pantoea* phage vB_PagM_LIET2 (242) | 42/45 (249) | 2e-32 |
| ORF25 (23077..23310) | AZS06265.1 structural protein (77) | no significant match | | |
| ORF26 complement (23338..23676) | AZS06266.1 putative transcriptional regulator (112) | ALJ98204.1 hypothetical protein *Klebsiella* phage vB_Kp3 (67) | 39/61 (67) | 6e-12 |
| ORF27 complement (23724..24215) | AZS06267.1 single-stranded DNA-binding protein (163) | YP_009845965.1 ssDNA-binding protein *Pantoea* phage vB_PagM_PSKM (176) | 59/64 (176) | 3e-73 |
| ORF28 complement (24219..24905) | AZS06268.1 recombinase (228) | ALJ98206.1 recombinase *Klebsiella* phage vB_Kp3 (231) | 41/44 (245) | 5e-47 |
| ORF29 complement (24902..25939) | AZS06269.1 putative exodeoxyribonuclease VIII (345) | QBZ71588.1 putative exonuclease *Escherichia* phage Sortsne (346) | 44/62 (327) | 4e-90 |
| ORF30 complement (25976..26455) | AZS06270.1 putative nuclease containing VRR-NUC domain (159) | YP_009816879.1 VRR-NUC nuclease *Escherichia* phage Skarpretter (139) | 53/55 (99) | 1e-14 |
| ORF31 complement (26455..28407) | AZS06271.1 DNA helicase (650) | ALJ98210.1 DNA helicase *Klebsiella* phage vB_Kp3 (682) | 53/67 (680) | 0.0 |
| ORF32 (28477..29124) | AZS06272.1 DNA N-6-adenine-methyltransferase (215) | YP_006383647.1 N-6-adenine-methyltransferase *Xanthomonas* phage vB_XveM_DIBBI (237) | 39/52 (235) | 1e-37 |
| ORF33 (29167..29253) | AZS06273.1 hypothetical protein (28) | no significant match | | |
| ORF34 (29266..29631) | AZS06274.1 hypothetical protein (121) | AEM24720.1 hypothetical protein *Cronobacter* phage ES2 (85) | 46/64 (82) | 4e-18 |
| ORF35 (29628..29834) | AZS06275.1 hypothetical protein (68) | no significant match | | |
| ORF36 (29834..30043) | AZS06276.1 hypothetical protein (69) | ATI16447.1 hypothetical protein *Klebsiella* phage vB_KpnS_IME279 (75) | 40/57 (73) | 6e-10 |
| ORF37 (30043..30333) | AZS06277.1 hypothetical protein (96) | ATI16408.1 hypothetical protein *Klebsiella* phage vB_KpnS_IME279 (86) | 56/66 (36) | 3e-07 |
| ORF38 (30333..31118) | AZS06278.1 preQ$_0$ DNA deoxyribosyltransferase (DpdA) (261) | YP_009217891.1 hypothetical protein SEN1_38 *Salmonella* phage SEN1 (278) | 54/67 (265) | 7e-102 |
| ORF39 (31231..31536) | AZS06279.1 hypothetical protein (101) | no significant match | | |
| ORF40 (31544..31849) | AZS06280.1 hypothetical protein (101) | no significant match | | |
| ORF41 (31849..32436) | AZS06281.1 putative DNA-binding protein (195) | hypothetical protein DIBBI_gp76 *Xanthomonas* phage vB_XveM_DIBBI (116) | 35/58 (89) | 3e-07 |
| ORF42 (32482..32482) | AZS06282.1 hypothetical protein (112) | no significant match | | |
| ORF43 (32810..33058) | AZS06283.1 hypothetical protein (82) | no significant match | | |

| | | | | |
|---|---|---|---|---|
| ORF44 (33058..33588) | AZS06284.1 hypothetical protein (176) | YP_009818377.1 hypothetical protein AAS23_gp75 *Pantoea* phage vB_PagS_AAS23 (151) | 45/57 (133) | 5e-23 |
| ORF45 (33585..33857) | AZS06285.1 hypothetical protein (90) | no significant match | | |
| ORF46 (33854..34495) | AZS06286.1 hypothetical protein (213) | YP_009168932.1 conserved phage protein *Escherichia* phage vB_EcoM_ECO1230-10 | 36/54 (223) | 3e-32 |
| ORF47 (34605..34922) | AZS06287.1 hypothetical protein (105) | no significant match | | |
| ORF48 (34919..35263) | AZS06288.1 hypothetical protein (114) | no significant match | | |
| ORF49 (35256..35804) | AZS06289.1 inner-membrane spanin protein Rz (182) | YP_009284328.1 hypothetical protein BI096_gp33 *Enterobacter* phage Arya (177) | 45/58 (168) | 2e-23 |
| ORF50 (35428..35742) | AZS06290.1 outer-membrane spanin protein Rz1 (105) | AXC42977.1 O-spanin *Salmonella* phage Skate (88) | 33/54 | 0.001 |
| ORF51 (35782..36297) | AZS06291.1 endolysin (171) | YP_008239475.1 lysozyme *Salmonella* phage FSL SP-058 (163) | 55/67 (174) | 1e-55 |
| ORF52 (36294..36524) | AZS06292.1 hypothetical protein (76) | no significant match | | |
| ORF53 complement (36616..37392) | AZS06293.1 hypothetical protein (258) | YP_009843749.1 hypothetical protein HWC07_gp068 *Pantoea* phage vB_PagM_LIET2 (131) | 45/56 (75) | 5e-13 |
| ORF54 complement (37402..37632) | AZS06294.1 hypothetical protein (76) | no significant match | | |
| ORF55 complement (37619..37840) | AZS06295.1 hypothetical protein (73) | no significant match | | |
| ORF56 complement (37827..38231) | AZS06296.1 hypothetical protein (134) | no significant match | | |
| ORF57 complement (38240..38626) | AZS06297.1 hypothetical protein (128) | no significant match | | |
| ORF58 complement (38671..38886) | AZS06298.1 hypothetical protein (71) | YP_009284319.1 hypothetical protein BI096_gp42 *Enterobacter* phage Arya (71) | 82/91 (71) | 1e-36 |
| ORF59 complement (38859..39029) | AZS06299.1 hypothetical protein (56) | no significant match | | |
| ORF60 complement (39060..39473) | AZS06300.1 hypothetical protein (137) | no significant match | | |
| ORF61 complement (39525..39848) | AZS06301.1 hypothetical protein (107) | ORM93120.1 hypothetical protein HA50_07085 *Pantoea cypripedii* (174) | 45/50 (98) | 4e-10 |
| ORF62 complement (39896..40798) | AZS06302.1 adhesin (300) | QAX92379.1 adhesin *Pantoea* phage vB_PagM_LIET2 (425) | 36/57 (214) | 1e-23 |
| ORF63 complement (40885..41109) | AZS06303.1 hypothetical protein (74) | no significant match | | |

| | | | | |
|---|---|---|---|---|
| ORF64 complement (41121..41366) | AZS06304.1 hypothetical protein (81) | QAX92261.1 hypothetical protein LIET2_gp009 *Pantoea* phage vB_PagM_LIET2 (80) | 84/85 (81) | 2e-63 |
| ORF65 complement (41363..41536) | AZS06305.1 hypothetical protein (57) | no significant match | | |
| ORF66 complement (41533..41883) | AZS06306.1 hypothetical protein (116) | YP_009638919.1 hypothetical protein H66_004 *Pseudomonas* virus H66 (393) | 48/50 (102) | 5e-24 |
| ORF67 complement (41880..41996) | AZS06307.1 hypothetical protein (38) | no significant match | | |
| ORF68 (42066..44777) | AZS06308.1 putative DNA primase/replicative helicase (903) | QHR72895.1 hypothetical protein sortsyn_30 *Escherichia* phage sortsyn (562) | 43/46 (332) | 4e-48 |
| ORF69 (44758..44859) | AZS06309.1 hypothetical protein (33) | no significant match | | |
| ORF70 (44865..45011) | AZS06310.1 hypothetical protein (48) | no significant match | | |
| ORF71 (44980..45174) | AZS06311.1 hypothetical protein (64) | no significant match | | |
| ORF72 (45282..45809) | AZS06312.1 hypothetical protein (175) | no significant match | | |
| ORF73 (45806..46012) | AZS06313.1 hypothetical protein (68) | no significant match | | |

**Table S3.** Structural MED16 proteins identified by MS.

| Gene | Putative function | MW (KDa) | Peptide count | Sequence coverage (%) |
|---|---|---|---|---|
| ORF21 | tail fiber protein | 119.461 | 19 | 19.89 |
| ORF16 | tape measure protein | 92.519 | 62 | 55.22 |
| ORF23 | tail fiber protein | 79.727 | 10 | 12.33 |
| ORF03 | portal protein | 55.853 | 2 | 3.88 |
| ORF04 | head morphogenesis protein | 40.625 | 18 | 53.53 |
| ORF08 | major capsid protein | 39.511 | 17 | 32.71 |
| ORF13 | major tail protein | 25.262 | 1 | 4.64 |
| ORF11 | neck protein | 24.910 | 12 | 34.38 |
| ORF24 | structural protein | 24.392 | 6 | 31.66 |
| ORF51 | endolysin | 18.831 | 5 | 32.16 |
| <u>ORF25</u> | structural protein | 7.866 | 1 | 16.88 |

MED16 specific ORF with no reliable identity to database entries is underlined.

**Table S4**. Top matches for BLAST-based alignments of whole genome sequences of MED16 and its closest relatives generated using PASC.

| The overall nucleotide sequence identity (%) | Reference Sequence | Bacteriophage | Genus |
|---|---|---|---|
| 25.98% | MT075871.1 | *Klebsiella* phage vB_KleS-HSE3 | unclassified *Siphoviridae* |
| 25.46% | KT367887.1 | *Klebsiella* phage vB_Kp3 | unclassified *Siphoviridae* |
| 25.06% | MN656993.1 | *Enterobacter* phage ATCEA85 | unclassified *Siphoviridae* |
| 23.32% | NC_042029.1 | *Edwardsiella* phage eiAU | *Eiauvirus* |
| 23.23% | NC_023555.1 | *Edwardsiella* phage eiAU-183 | *Eiauvirus* |
| 23.09% | NC_019419.2 | *Escherichia* virus JL1 | *Dhillonvirus* |
| 22.97% | NC_028901.1 | *Escherichia* phage slur05 | *Dhillonvirus* |
| 22.91% | NC_031081.1 | *Escherichia* phage Envy | *Dhillonvirus* |
| 22.75% | NC_016566.1 | *Shigella* phage EP23 | *Dhillonvirus* |
| 22.70% | NC_019724.1 | *Escherichia* phage HK578 | *Dhillonvirus* |

**Table S5.** A list of *Pantoea* bacteriophages with completely sequenced genomes, that have been published and/or deposited in the public databases.

| Phage | GenBank accession no. | Family | Genome size (bp) | Reference |
|---|---|---|---|---|
| Phynn | MN038175.1 | *Myoviridae* | 173720 | unpublished |
| vB_PagM_LIET2 | MK388689.1 | *Myoviridae* | 74710 | unpublished |
| Kyle | NC048796.1 | *Myoviridae* | 73168 | unpublished |
| vB_PagM_SSEM1 | MT230534.1 | *Myoviridae* | 54982 | unpublished |
| vB_PagM_AAM37 | MK798143.1 | *Myoviridae* | 49990 | unpublished |
| vB_PagM_PSKM | MK798144.1 | *Myoviridae* | 49935 | unpublished |
| vB_PagM_AAM22 | MK798142.1 | *Myoviridae* | 49744 | [4] |
| vB_PagS_AAS21 | MK770119.1 | *Siphoviridae* | 116649 | [5] |
| vB_PagS_Vid5 | MG948468.1 | *Siphoviridae* | 61437 | [3] |
| vB_PagS_AAS23 | MK095606.1 | *Siphoviridae* | 51170 | [6] |
| **vB_PagS_MED16** | **MK095605.1** | ***Siphoviridae*** | **46103** | **this study** |
| LIMElight | FR687252.1 | *Podoviridae* | 44546 | [7] |
| LIMEzero | FR751545.1 | *Podoviridae* | 43032 | [7] |
| vB_PagP-SK1 | MN450150.1 | *Podoviridae* | 39938 | [8] |

**Table S6:** DpdA homologues extracted from HMMER searches against nr90_15_Jan database at MPI Bioinformatics Toolkit. The results were supplemented with phage DpdA sequences from (PMID: 31784519) and the resulting set of sequences was clustered and sequence groups were identified with CLANS. Supplementary material is provided in a separate file (**Table S6**).

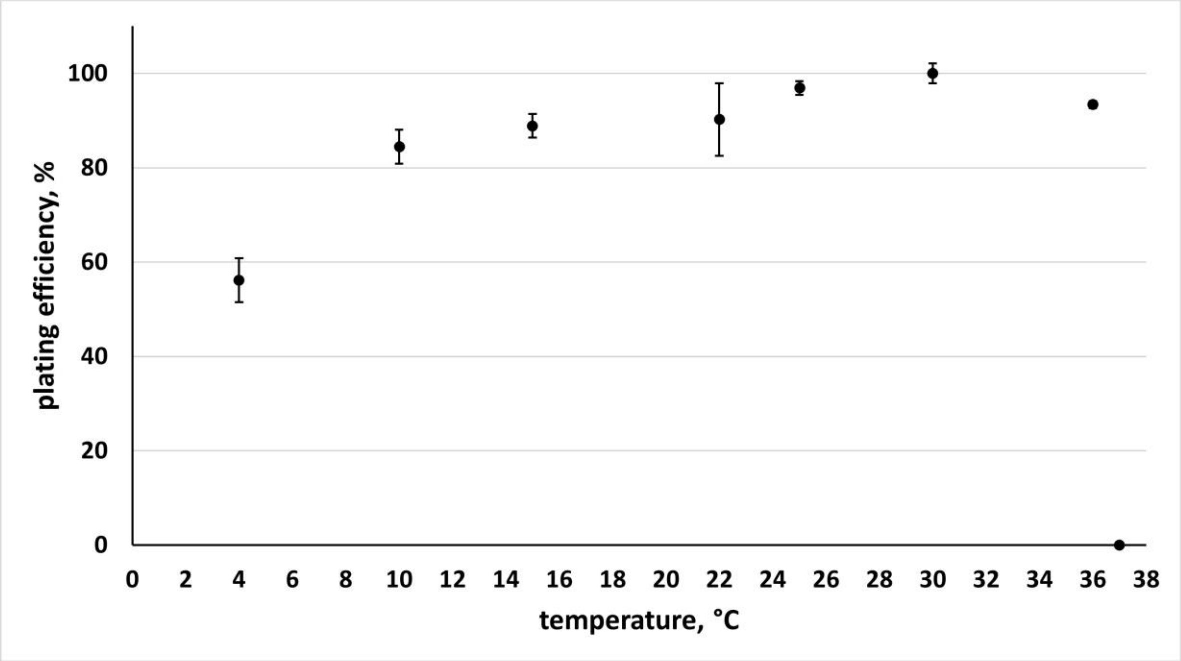


**Figure S1.** Effect of temperature on the efficiency of plating of phage MED16. Each point represents the mean of three independent experiments. Error bars indicate standard deviation.

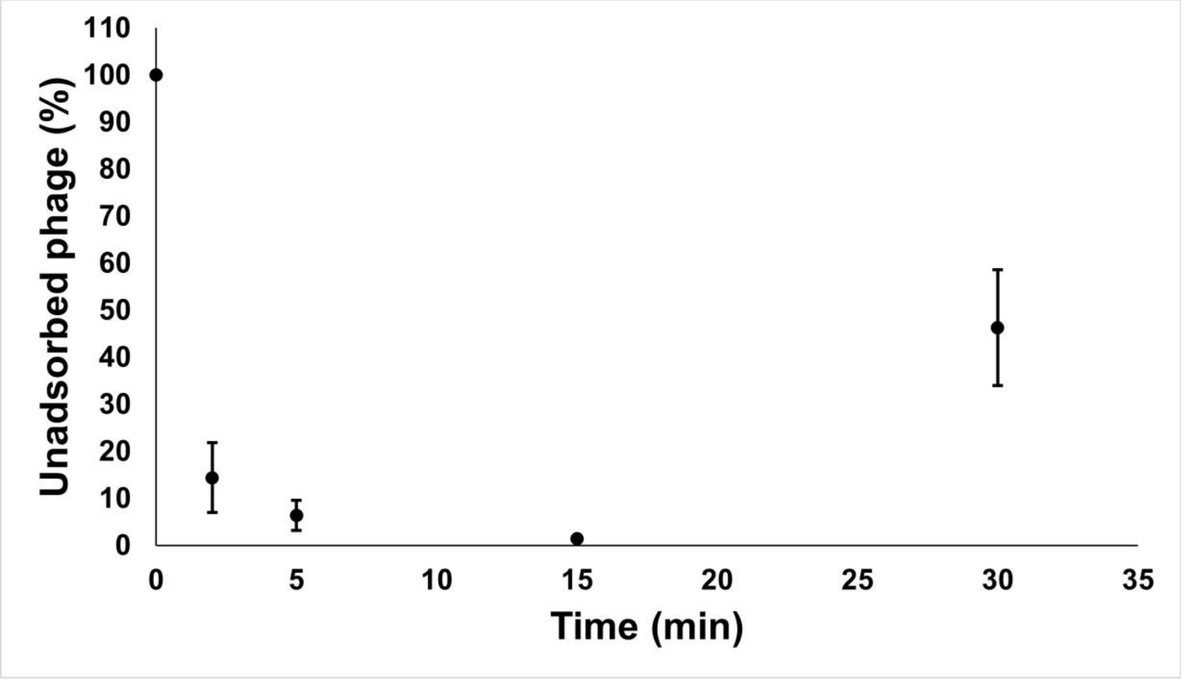


**Figure S2.** Adsorption rate of phage MED16. Each point represents the mean of three independent experiments. Error bars indicate standard deviation.

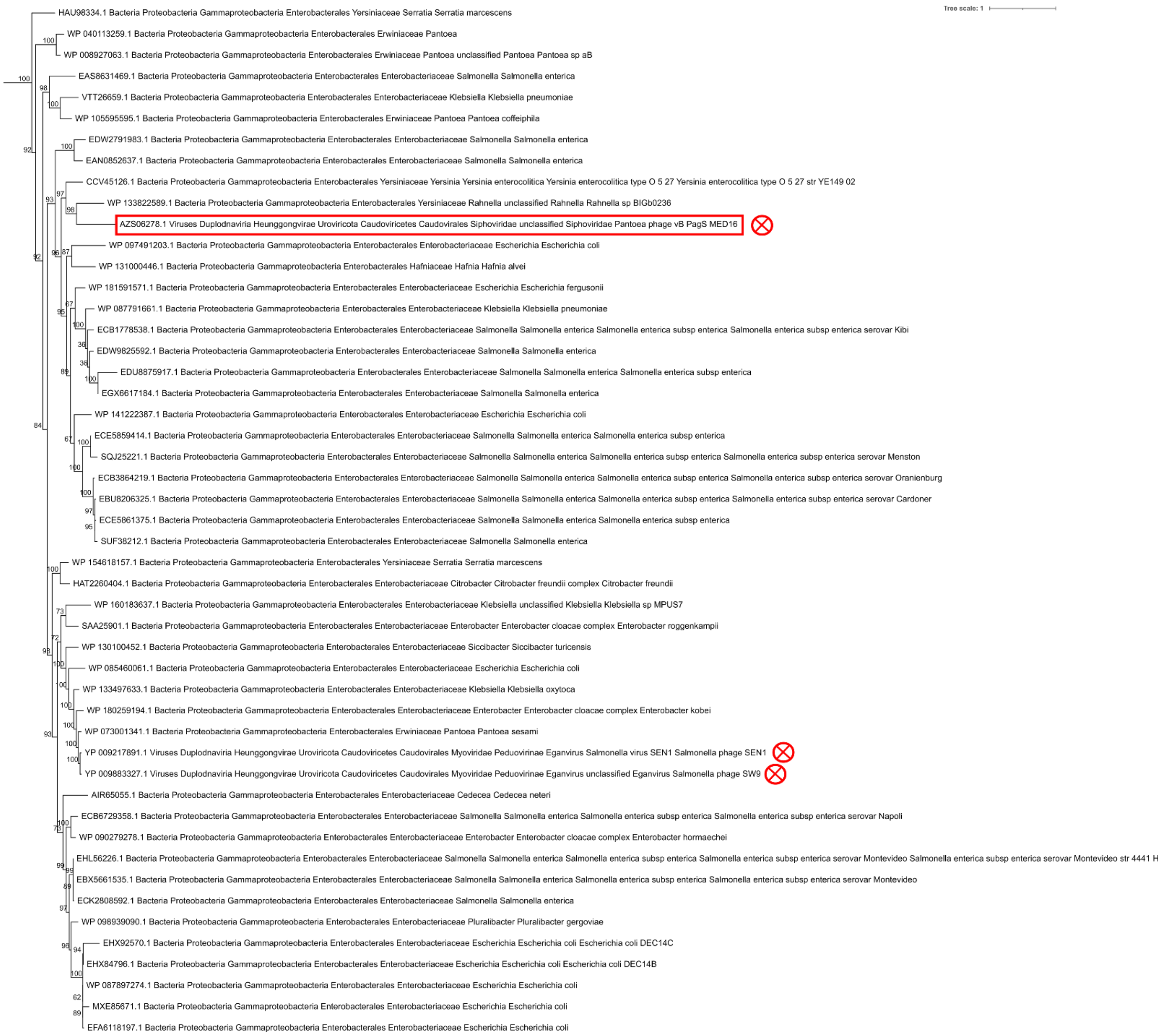

**Figure S3.** A branch of a DpdA-like phylogenetic tree containing DpdA from phage MED16. ModelFinder has selected the “LG+R10” model of evolution as best-fit for our alignment. MED16 DpdA viral homologues are marked. The percentage of replicate trees in which the associated taxa clustered together in the bootstrap test is shown next to the branches.

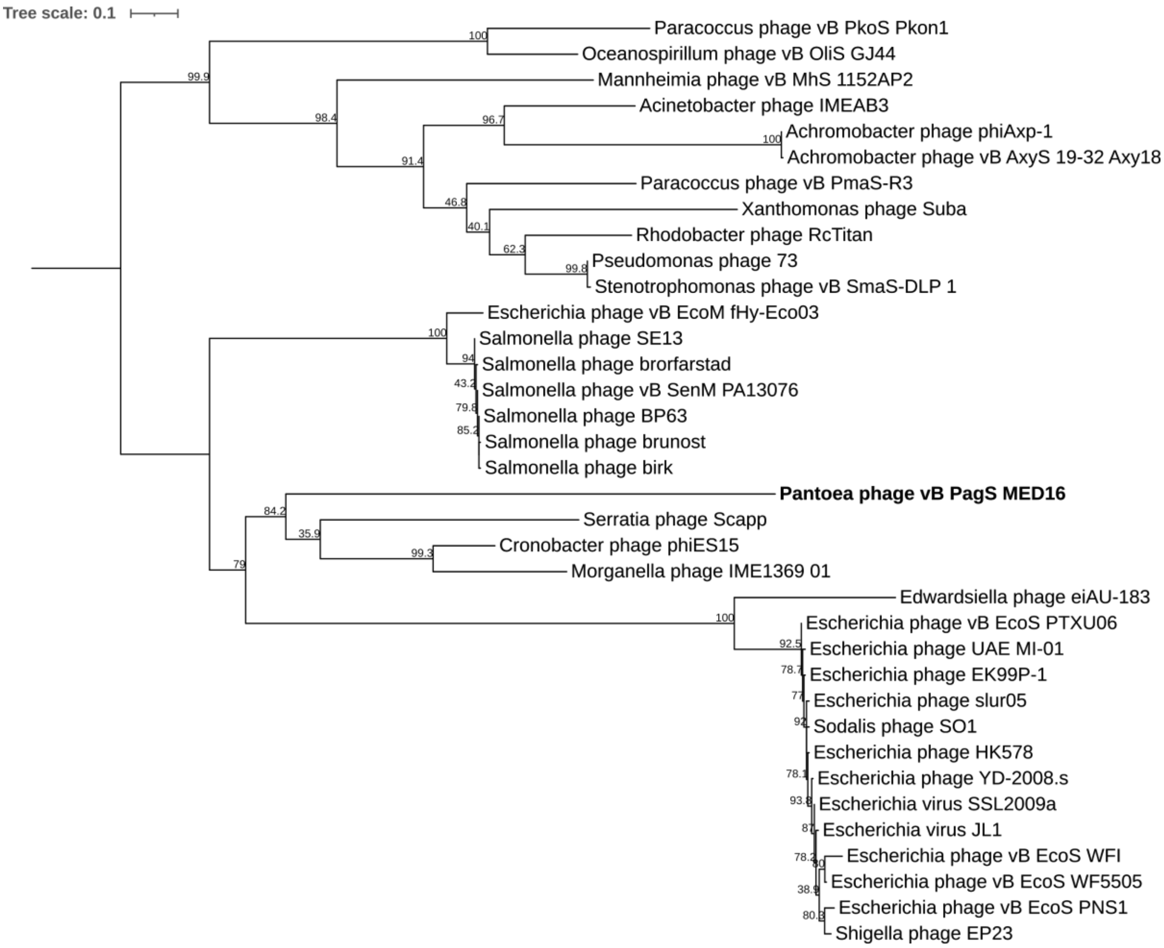


**Figure S4**. A branch of a major capsid protein phylogenetic tree containing major capsid protein from phage MED16. ModelFinder has selected the “LG+F+I+G4” model of evolution as best-fit for our alignment. The percentage of replicate trees in which the associated taxa clustered together in the bootstrap test is shown next to the branches.

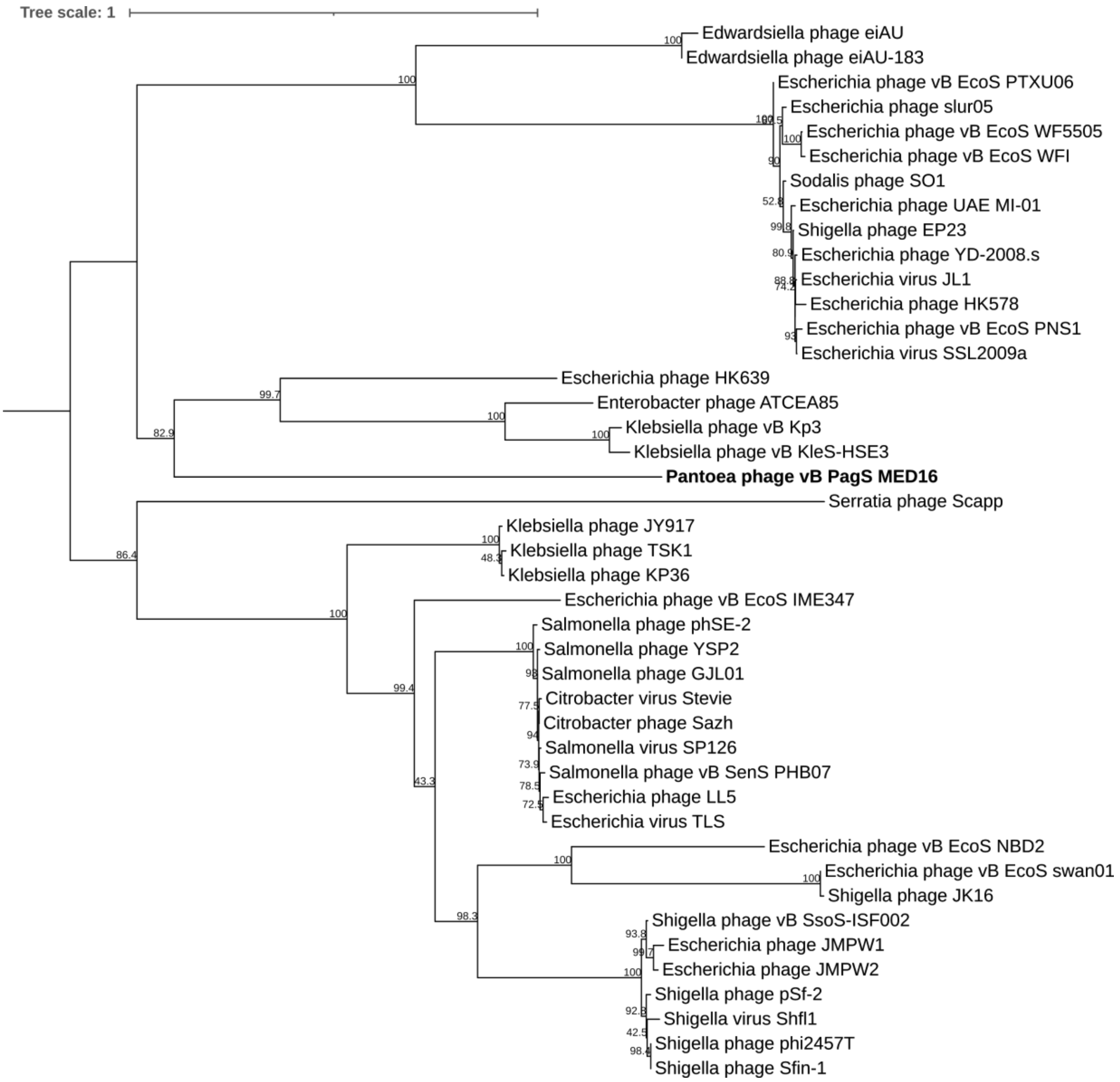


**Figure S5.** A branch of a tape measure protein phylogenetic tree containing tape measure protein from phage MED16. ModelFinder has selected the "LG+F+R3" model of evolution as best-fit for our alignment. The percentage of replicate trees in which the associated taxa clustered together in the bootstrap test is shown next to the branches.

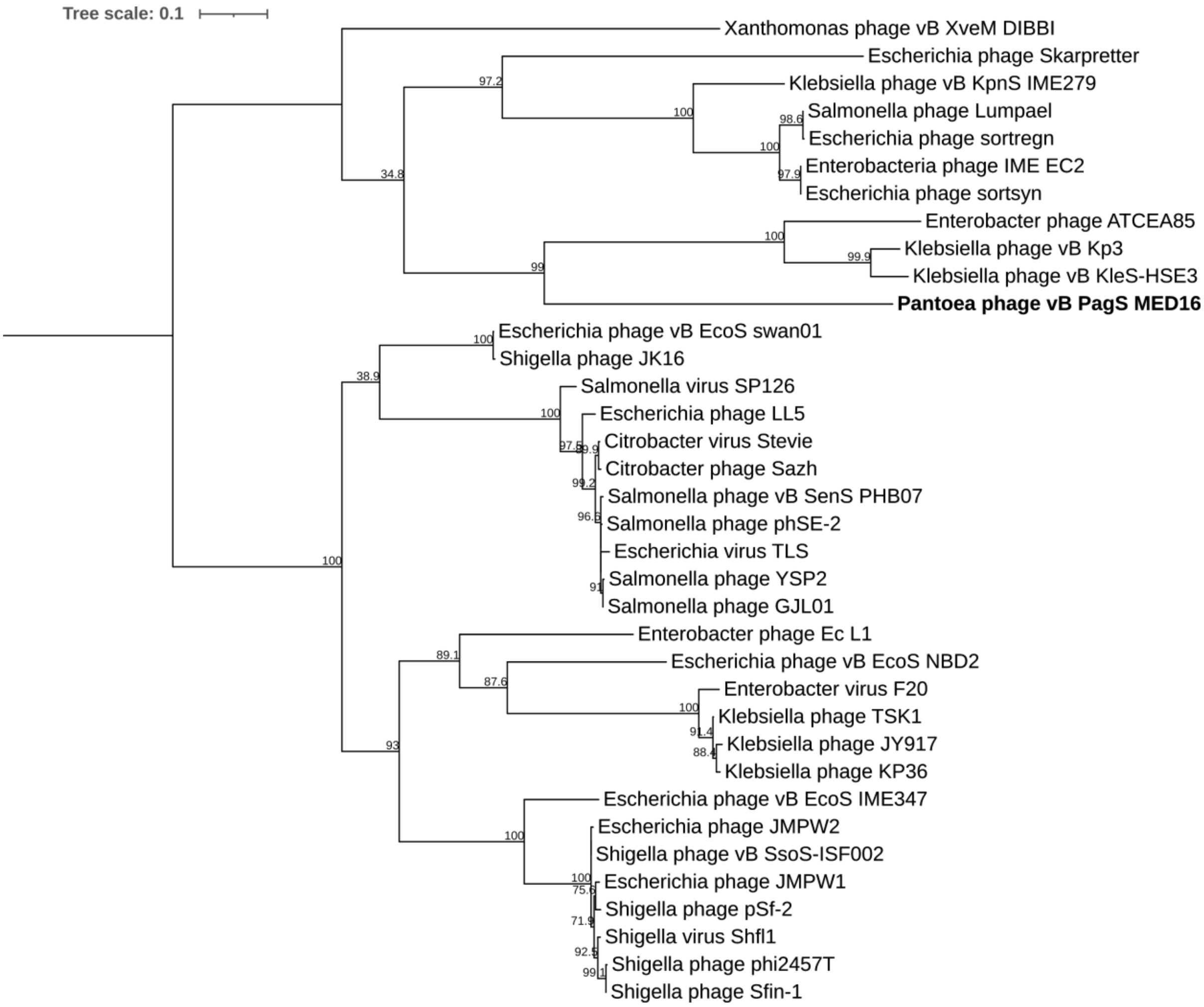


**Figure S6**. A branch of a helicase phylogenetic tree containing helicase from phage MED16. ModelFinder has selected the "WAG+F+I+G4" model of evolution as best-fit for our alignment. The percentage of replicate trees in which the associated taxa clustered together in the bootstrap test is shown next to the branches.

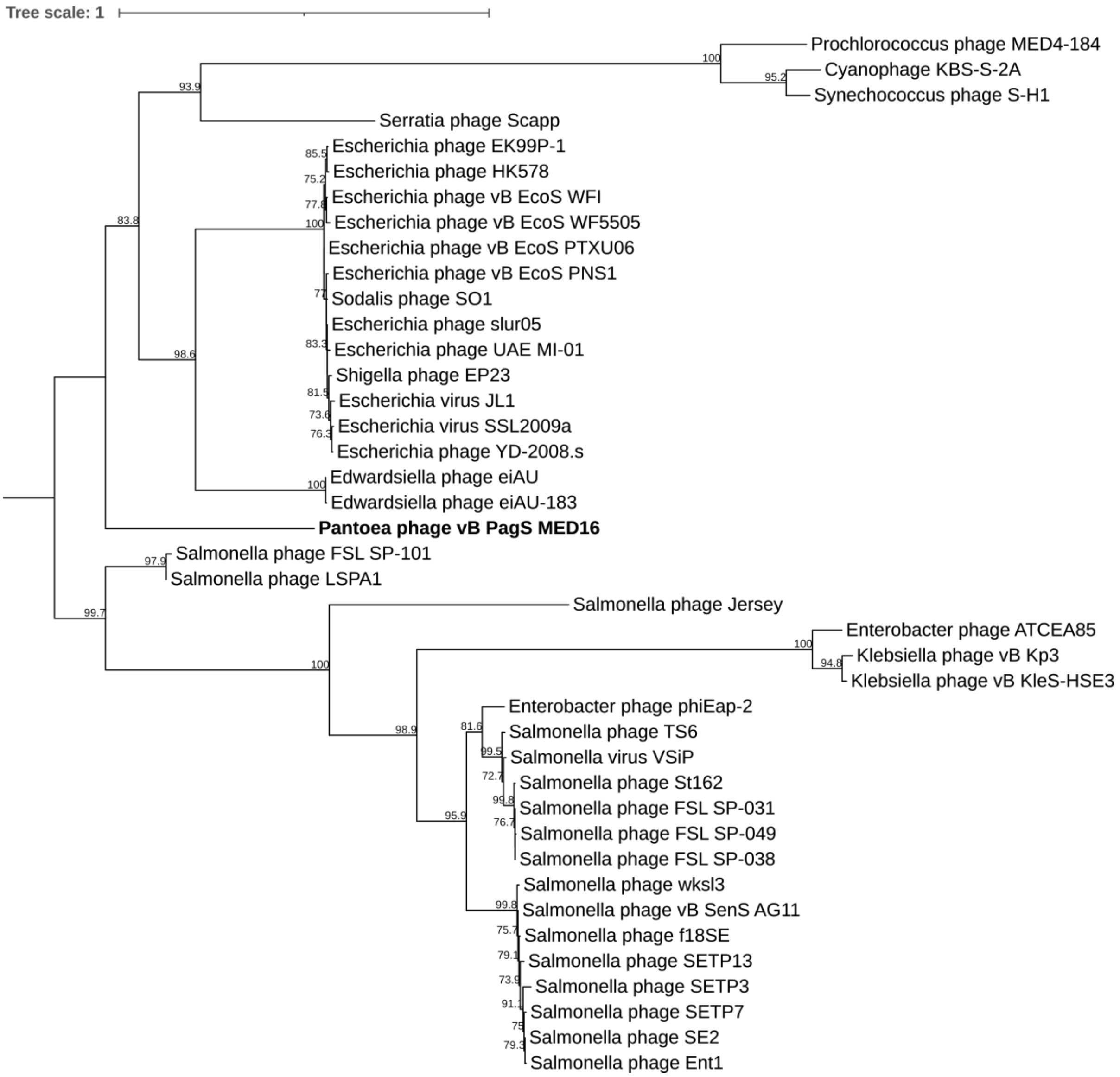


**Figure S7**. A branch of a terminase large subunit phylogenetic tree containing terminase large subunit from phage MED16. ModelFinder has selected the "LG+I+G4" model of evolution as best-fit for our alignment. The percentage of replicate trees in which the associated taxa clustered together in the bootstrap test is shown next to the branches.

## Supplementary references


1. Hyman, P.; Abedon, S.T. Bacteriophage host range and bacterial resistance. In Advances in Applied Microbiology; Laskin, A.I., Sariaslani, S., Gadd, G.M., Eds.; Elsevier Inc. Academic Press: Burlington, MA, USA, 2010; Volume 70, pp. 217–248.
2. Šimoliūnas, E.; Kaliniene, L.; Truncaitė, L.; Zajančkauskaitė, A.; Staniulis, J.; Kaupinis, J.; Ger, M.; Valius, M.; Meškys, R. *Klebsiella* phage vB_KleM-RaK2 – a giant singleton virus of the family *Myoviridae*. *PLoS ONE* **2013**, *8*, e60717.
3. Šimoliūnas, E.; Šimoliūnienė, M.; Kaliniene, L.; Zajančkauskaitė, A.; Skapas, M.; Meškys, R.; Kaupinis, A.; Valius, M.; Truncaitė, L. Pantoea bacteriophage vB_PagS_Vid5: a low-temperature siphovirus that harbors a cluster of genes involved in the biosynthesis of archaeosin. Viruses 2018, 10, 583.
4. Truncaitė, L.; Šimoliūnienė, M.; Alijošius, L.; Petrauskaitė, E.; Kiaušaitė, L.; Meškys, R.; Skapas, M.; Šimoliūnas, E. Complete genome analysis of *Pantoea agglomerans*-infecting bacteriophage vB_PagM_AAM22. *Arch. Virol*. **2020**, *165*, 2111–2114

5. Šimoliūnienė, M.; Truncaitė, L.; Petrauskaitė, E.; Zajančkauskaitė, A.; Meškys, R.; Skapas, M.; Kaupinis A.; Valius M.; Šimoliūnas, E. *Pantoea agglomerans*-infecting bacteriophage vB_PagS_AAS21: a cold-adapted virus representing a novel genus within the family *Siphoviridae*. *Viruses* **2020**, *12*, 479.
6. Žukauskienė E, Šimoliūnienė M, Truncaitė L, Skapas M, Kaupinis A, Valius M, Meškys R, Šimoliūnas E. Pantoea Bacteriophage vB_PagS_AAS23: A Singleton of the Genus Sauletekiovirus. *Microorganisms* **2021**, *9*, 668.
7. Adriaenssens, E.M.; Ceyssens, P.J.; Dunon, V.; Ackermann, H.W.; Van Vaerenbergh, J.; Maes, M.; De Proft, M.; Lavigne, R. Bacteriophages LIME-light and LIMEzero of *Pantoea agglomerans*, belonging to the "phiKMV-like viruses". *Appl. Environ. Microbiol.* **2011**, 77, 3443–3450.
8. McDougall, D.L.; Soutar, C.D.; Perry, B.J.; Brown, C.; Alexander, D.; Yost, C.K.; Stavrinides, J. Isolation and characterization of vB_PagP-SK1, a T7-like phage infecting *Pantoea agglomerans*. *J. PHAGE* **2020**, *1*, 45–56.
